# Modeling, environmental and economic analysis of drying of orange slice in an automatic indirect mixed mode solar dryer

**DOI:** 10.1038/s41598-025-29436-x

**Published:** 2026-01-28

**Authors:** Abdallah Elshawadfy Elwakeel, Awad Ali Tayoush Oraiath, Wajdi Aissa Mohammed Abdurraziq, Ahmed Elbeltagi, András Székács, Omar Saeed, Mohamed Hamdy Eid, Mohammad S. AL-Harbi, Atef Fathy Ahmed, Aml Abubakr Tantawy

**Affiliations:** 1 Agricultural Engineering Department, Faculty of Agriculture and Natural Resources, Aswan University, Aswan, 81528 Egypt; 2 Department of Agricultural Engineering, Faculty of Agriculture, Omar Al Mukhtar University, P.O. Box 991, Al Bayda, Libya; 3 Agronomy Department, Faculty of Agriculture, Omar Al Mukhtar University, P.O. Box 991, Al Bayda, Libya; 4 Agricultural Engineering Department, Faculty of Agriculture, Mansoura University, Mansoura, 35516 Egypt; 5 Agro-Environmental Research Centre, Institute of Environmental Sciences, Hungarian University of Agriculture and Life Sciences, Páter Károly u. 1, Gödöllő, 2100 Hungary; 6 Doctoral School of Environmental Science, Hungarian University of Agriculture and Life Sciences (MATE), Páter Károly u. 1, Gödöllő, 2100 Hungary; 7 Institute of Environmental Management, Faculty of Earth Science, University of Miskolc, Miskolc- Egyetemváros, 3515 Hungary; 8 Geology Department, Faculty of Science, Beni-Suef University, Beni-Suef, 65211 Egypt; 9 Department of Biology, College of Science, Taif University, P.O. Box 11099, Taif, 21944 Saudi Arabia; 10 Food Science Department, Faculty of Agriculture, Beni-Suef University, Beni-Suef, 65211 Egypt

**Keywords:** Fruit drying, Renewable energy, Sustainable agriculture, Thin-layer modeling, Drying kinetics, Energy science and technology, Engineering, Environmental sciences

## Abstract

This study presents an indirect mixed-mode solar dryer (IMMSD) integrated with a photovoltaic system with an automatic, temperature-responsive control system that automatically switches between natural and forced convection, enhancing efficiency and reducing energy use. Unlike previous fixed systems, it prevents over-drying and spoilage. The cost-effective, solar-powered design suits off-grid communities. By integrating drying kinetics with economic and environmental assessments, the system supports sustainability goals. The study also examines how slice thicknesses and tray positions within the drying room affect orange drying kinetics. Then, the IMMSD was used to dry orange slices under real conditions at Aswan University, Egypt, in January 2025, aiming to evaluate performance across different slice thicknesses (4, 6, and 8 mm) and tray positions (lower, middle, and upper levels). The results demonstrated that orange slices with a 4 mm thickness dried on the lower tray reached the final moisture content (MC) more quickly than thicker slices (8 mm) dried on the middle and upper trays. The 4 mm slices achieved the lowest final MC at approximately 12.5%, with a 48% reduction in drying time compared to 8 mm slices on the upper tray. The effective moisture diffusivity (D_eff_) ranged from 4.5 × 10⁻⁸ to 15 × 10⁻⁸ m²/s. Additionally, five semi-theoretical models—Midilli, Modified Midilli I and II, Aghbashlo, and Henderson–Pabis—provided the best fit for modeling the drying behavior of orange slices. The environmental analysis showed that the energy required for moisture evaporation ($\:{E}_{at}$) increased with slice thickness, reaching 916.56, 1372.31, and 1839.76 kWh for 4, 6, and 8 mm slices, respectively. The 8 mm slices yielded the highest annual dried product output, net CO₂ reduction (90.72 tons), and the shortest energy payback time (0.85 years). Carbon credits ranged from 2179.29 to 4535.76 USD. Economically, the IMMSD required a low capital cost of 700 USD and annual costs of 996.12 USD, generating yearly savings of up to 14,015.9 USD, that reduced the payback period of investigation up to only about one month.

## Introduction

Oranges are among the most popular and widely consumed fruits globally, valued for their unique taste, nutritional richness, and broad health benefits. Their importance extends beyond nutrition, as they are also used in traditional medicine and the food industry^1,2^. Oranges are an excellent source of vitamin C, dietary fiber, and a variety of B vitamins. They also contain important phytochemicals such as carotenoids, flavonoids, and limonoids, which possess antioxidant properties and contribute to overall health^3,4^. Regular consumption of oranges has been linked to a reduced risk of chronic diseases, as their nutrients and bioactive compounds help prevent cardiovascular diseases, lower cholesterol levels, and support gastrointestinal health . In addition, oranges and their juices support immune function, reduce inflammation, and help manage oxidative stress—effects primarily attributed to vitamin C and polyphenols like hesperidin and naringin. Human studies have demonstrated that orange juice can significantly lower markers of inflammation^7^. Furthermore, oranges exhibit a wide range of medicinal properties, including anti-bacterial, anti-fungal, anti-diabetic, cardio-protective, anti-cancer, anti-arthritic, anti-inflammatory, antioxidant, and anti-hypertensive effects^5,6^.

Egypt is a leading producer of oranges, with production averaging around 2.3 million tons annually between 1997 and 2019, reaching a maximum of 3.35 million tons in some years^8^. Oranges are the most important citrus crop in Egypt, accounting for approximately 72.2% of total citrus production in 2019/2020. The main cultivated variety is the Washington navel orange, which is favored for its taste, nutrition, and seedless nature^9^. The area dedicated to fruitful orange cultivation averaged about 247,010 acres from 2000 to 2022, with production supporting both local consumption and significant exports^2^. Egypt has become the world’s largest exporter of oranges, with export earnings reaching $661 million in 2019/2020^9^. Globally, oranges are one of the most widely produced and consumed fruits. Egypt is among the top producers and exporters, with its oranges reaching markets in Russia, Saudi Arabia, China, the Netherlands, Bangladesh, and India^2^. The global orange market is highly competitive, and Egypt’s large surplus allows it to play a significant role in international trade^2,10^.

Oranges are highly perishable and prone to quality deterioration and nutrient loss during storage, particularly under unsuitable conditions^11,12^. To address this challenge, solar dryers offer a sustainable, energy-efficient, and cost-effective preservation solution^13–16^. By utilizing renewable solar energy, these systems significantly enhance the drying speed while maintaining the fruit’s nutritional integrity and sensory attributes, such as color, aroma, and flavor^17–20^. Unlike traditional sun drying, solar dryers provide controlled drying conditions that reduce microbial contamination, minimize oxidation, and extend shelf life^21–23^. Additionally, their environmentally friendly design reduces dependence on fossil fuels and lowers greenhouse gas emissions, aligning with sustainable agricultural and food processing practices. These advantages make solar drying an ideal method for preserving oranges and other high-moisture fruits, especially in regions with abundant solar resources^24,25^.

Previous research investigating the drying of orange fruits using a variety of drying systems has provided valuable insights into their effects on drying efficiency, nutrient retention, and overall product quality. These studies can be broadly summarized as follows: Bozkir et al.^26^ investigated the effects of hot air, vacuum infrared, and vacuum microwave drying methods on orange slice quality and drying kinetics. Their findings highlighted vacuum microwave drying (VMD) as the most efficient technique, offering the fastest drying rates while best preserving phenolic compounds, vitamin C, and color—demonstrating superior performance in both quality retention and efficiency. Similarly, Özbek et al.^27^ explored hot air-assisted radio frequency drying (HA-RFD) of orange slices and concluded that this method significantly reduced drying time and enhanced phenolic, flavonoid, and antioxidant content compared to conventional hot air drying. The product quality achieved was comparable to freeze drying, positioning HA-RFD as a promising and innovative alternative. Nahar et al.^28^ examined the influence of various drying methods on orange peel quality attributes. Microwave drying at 600 W improved phenolic and flavonoid content, while freeze drying preserved β-carotene most effectively and resulted in the lightest, hardest texture. Among kinetic models, the Weibull model provided the best fit. Ramos et al.^29^ also evaluated orange peel quality under different drying techniques and found freeze drying to yield the best color and highest phenolic and antioxidant levels, whereas convective and microwave drying increased browning—reinforcing freeze drying as the preferred method for optimal quality preservation. Erten et al.^30^ compared tray drying, vacuum infrared drying (VID), and VMD on orange peels, showing that VMD provided the fastest drying and best retention of vitamin C, phenolics, and carotenoids. VID was more effective in preserving volatile compounds, while tray drying was the most detrimental to quality. Chabane et al.^31^ analyzed the performance of a solar drying chamber powered by a solar air collector and emphasized that moisture removal efficiency and energy performance were significantly influenced by airflow rate—highlighting the need to optimize mass flow rate for effective solar drying. Farahmandfar et al.^32^ assessed the impact of different drying methods on bitter orange (Citrus aurantium L.) peel waste. Freeze drying was found to be the most effective in preserving lightness, essential oil yield, phenolic content, and antioxidant and antibacterial properties. Similarly, Nemati et al.^33^ observed that freeze drying retained the highest levels of color, essential oil yield, phenolics, and antioxidant activity in Thomson navel orange peel, outperforming convective, vacuum, and microwave drying techniques. Mei et al.^34^ compared various drying technologies for brocade orange peels and concluded that freeze drying resulted in the most visually appealing product and highest nutrient preservation. However, microwave drying excelled in enhancing the bioavailability of phenolic compounds and ascorbic acid, suggesting that the choice between the two methods may depend on whether appearance or bioactive availability is prioritized in commercial applications. Lastly, Smaoui et al.^35^ studied the impact of radio frequency-assisted hot air drying (RF-HAD) on orange peel quality. This method significantly reduced drying time and improved key quality indicators such as color, polyphenols, carotenoids, and antioxidant content when compared to traditional hot air drying, establishing RF-HAD as a highly effective technique for maintaining nutritional and functional qualities.

Photovoltaic-thermal (PVT) solar dryers are advanced systems that integrate solar thermal collectors with photovoltaic panels to simultaneously generate heat and electricity, enabling efficient and sustainable food drying. Recent studies have focused on enhancing their performance through innovative designs and the incorporation of energy storage technologies. For instance, a novel PVT dryer equipped with sand-filled thermal energy storage (TES) was tested for drying Moringa leaves under varying airflow conditions, demonstrating improved efficiency^36^. Other developments include a hybrid PVT dryer with an evacuated tube collector designed for cassava, showing superior drying kinetics, energy savings, and product quality compared to open sun drying^37^. Mixed-mode and greenhouse-integrated PVT dryers have also been evaluated through MATLAB-based modeling and real-time validation, while machine learning techniques, such as artificial neural networks (ANN), and computational fluid dynamics (CFD) have been employed to optimize airflow and predict drying performance^38–41^. Some systems further enhance functionality by integrating heat pumps or thermoelectric generators (TEGs) to boost energy recovery and storage^42,43^. Environmentally, PVT dryers significantly reduce energy consumption, CO₂ emissions, and operating costs compared to conventional methods, with economic analyses indicating favorable payback periods of 2.98 to 3.51 years, making them suitable for small-scale and rural applications^44^.

Based on the reviewed literature, it is evident that there is a notable gap in research concerning the application of solar energy—particularly solar drying methods—for drying orange slices. While numerous studies have focused on advanced drying technologies such as vacuum microwave, freeze, and radio frequency-assisted drying, limited attention has been given to the use of solar dryers, despite their potential as a cost-effective, sustainable, and environmentally friendly alternative. Additionally, this study introduces an automatic IMMSD that integrates an automatic, temperature-responsive control algorithm capable of automatically shifting between natural and forced convection based on real-time temperature readings. In contrast to earlier systems that relied on fixed or manually adjusted airflow, this adaptive setup enhances drying efficiency, lowers energy use, and helps prevent over-drying and spoilage. The design features a fully automated, solar-powered control unit built into a cost-effective structure, making it an affordable and scalable solution—especially beneficial for off-grid farming communities. Additionally, the research uniquely merges drying kinetics modeling with thorough economic and environmental assessments, offering a comprehensive evaluation of system performance. This integrated approach supports global sustainability objectives by promoting renewable energy use and cutting greenhouse gas emissions. In sun-abundant countries like Egypt, the system presents a practical step forward in environmentally friendly orange drying. The study also examines how slice thicknesses and tray positions within the drying room affect orange drying kinetics.

## Materials and methods

### Experimental setup

**Fig. 1:**
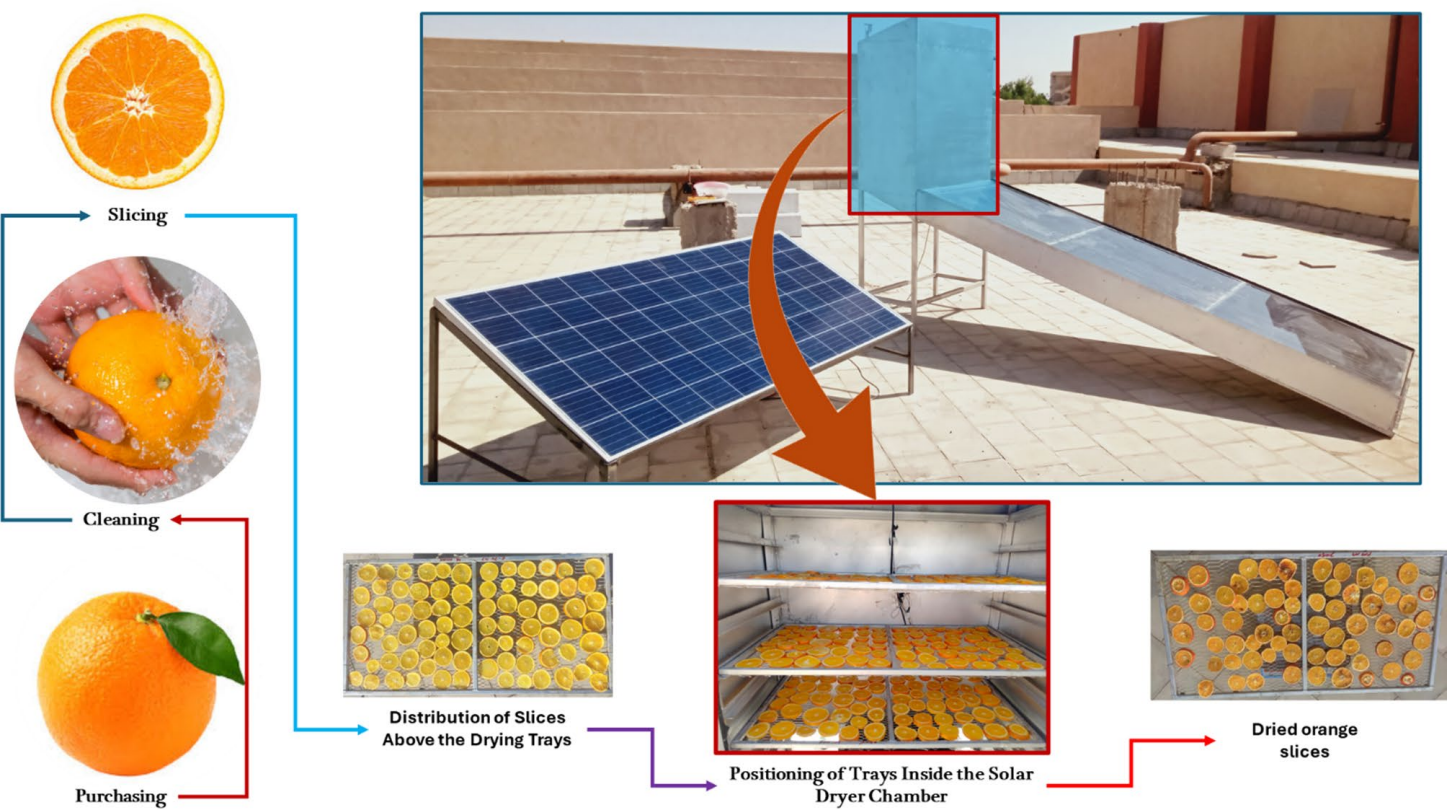
Process flow chart of orange preparation and drying using the IMMSD.

Fresh oranges were sourced from a local market in Aswan, Egypt, to ensure the availability of high-quality, locally grown produce. Upon procurement, the oranges were thoroughly washed with tap water to remove any adhering dust or surface impurities. The cleaned fruits were then manually sliced into uniform thicknesses of 4 mm, 6 mm, and 8 mm, in accordance with the slicing protocol described by Rokhbin and Azadbakht^45^, to investigate the influence of slice thickness on drying behavior. Following slicing, the orange slices were evenly and uniformly arranged on three separate drying trays to ensure consistent airflow and drying conditions, and the air flow rate was maintained at 0.13 m^3^/s. The trays were vertically spaced at intervals of approximately 35 cm to prevent airflow obstruction and facilitate efficient moisture removal. All drying experiments were conducted under controlled conditions at Aswan University during February 2025, taking advantage of the region’s high solar radiation levels. The drying process was carried out using an IMMSD. Figure 1 illustrates the process flow chart detailing the preparation of the orange slices and their subsequent drying within the IMMSD system. This standardized procedure ensured repeatability and reliability in analyzing the effects of slice thickness and tray position on drying kinetics and quality attributes.

### Description of the IMM

**Fig. 2:**
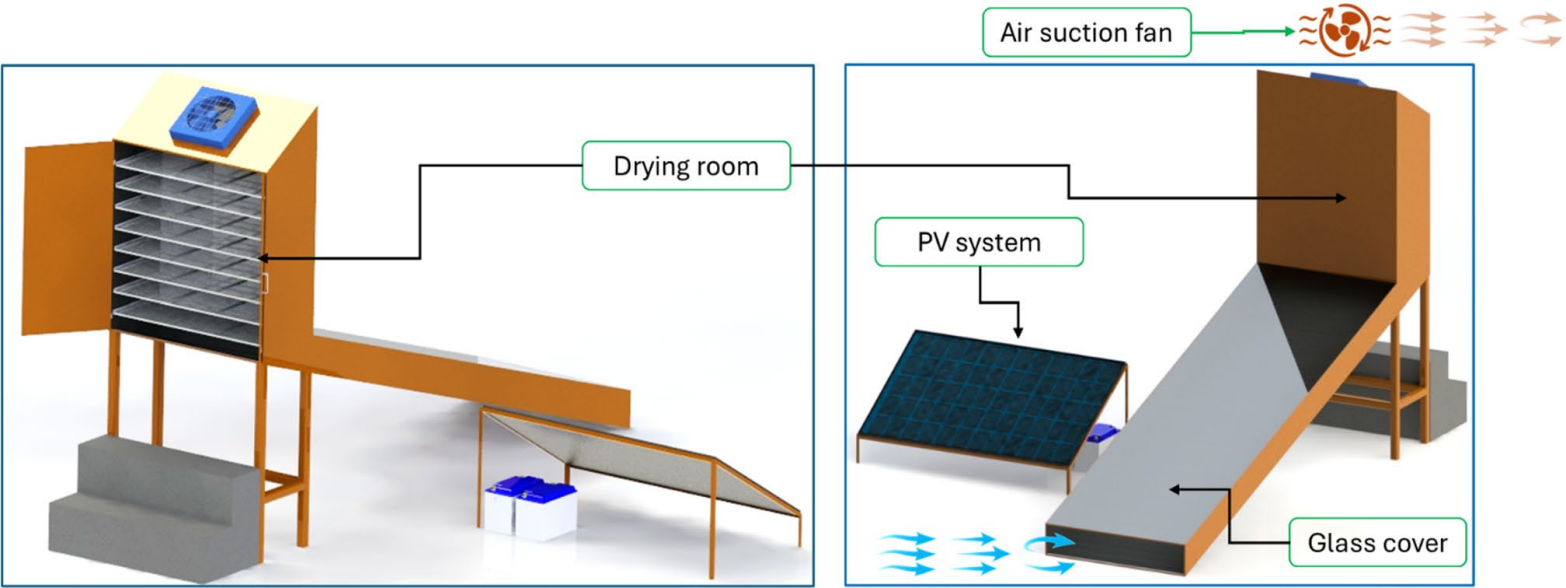
Main components of the IMMSD.

Figure 2 shows the main components of the IMMSD. Where the IMMSD consists of many parts including the flat plate solar collector, drying room, PV system, and measuring and control circuit. Table 1 shows the structural components and physical specifications of the Improved Mixed-Mode Solar Dryer (IMMSD), including the solar collector, drying chamber, drying trays, and airflow system. These elements constitute the main thermal and mechanical parts responsible for solar energy collection, heat transfer, and uniform air distribution during the drying process. Additionally, Table 2 shows the electronic, sensing, and control components integrated into the IMMSD, encompassing sensors, microcontroller units, communication modules, relay systems, and the photovoltaic power supply. These components enable automated monitoring, intelligent decision-making, and real-time control of the drying environment.

The control algorithm prioritizes energy efficiency by only activating components when necessary and includes a 5-minute loop delay for stable operation. This setup enables consistent, sustainable drying while preventing over-drying or spoilage. The system’s adaptability, low-cost components, and real-time monitoring capabilities make it an effective solution for solar drying in remote or energy-limited regions. In this study, the dryer operates in two clearly defined modes: (1) fan on, providing a constant airflow rate of 0.13 m³/s for forced convection, and (2) fan off, for natural convection.

**Table 1:** Structural components of the IMMSD.

Component	Specification	Description / Function
Solar Collector Type	Flat-plate solar air heater	Main source of heated air for drying
Collector Dimensions	300 × 100 × 20 cm	Provides large solar absorption area
Glazing Material	3 mm transparent glass	High solar transmittance; protects absorber
Absorber Plate	Corrugated black aluminum	High absorptivity surface for heat gain
Insulation	3 cm thermal wool	Reduces heat loss from back & sides
Drying Chamber Dimensions	100 × 50 cm	Enclosed drying space for trays
Drying Trays	3 trays used (designed for 8)	Spaced 35 cm vertically for uniform airflow
Airflow System	Four brushless DC fans (12 V)	Ensures uniform distribution of hot air
Operating Modes	(1) Forced convection: 0.13 m³/s (2) Natural convection	Optimizes energy use and drying performance

**Table 2:** Electronic, sensor, and control components of the IMMSD.

Electronic Component	Model / Specification	Function
Temperature & Humidity Sensors	Five DHT22 (AM2302, Adafruit, China)	Monitor drying conditions at inlet, outlet, and tray positions for control decisions
Light Intensity Sensor	GL5506 LDR (China)	Detects sunlight intensity for control decisions
Fan Speed Sensor	LM393 IR sensor (Waveshare, China)	Measures exhaust fan rotational speed for control decisions
Microcontroller Unit	Arduino Mega 2560 R3 (Italy)	The Arduino Mega serves as the central controller of the IMMSD, acquiring sensor data, executing the control algorithm, and automatically managing airflow and system operation to maintain optimal drying conditions.
Relay Module	Songle SRD-05VDC-SL-C (2-channel)	Switches DC fans based on set thresholds
Communication Module	SIM900A GSM (SIMCom, China)	Sends SMS alerts for system status/faults
User Interface	16 × 2 LCD display	Shows temperature, humidity, and system state
Power System	PV panel: 320 W polycrystalline (CS6X-320P)	Supplies off-grid power to all components
Charge controller	RBL-30 A	
Battery	12 V, 70 A	

### Evaluation of the drying kinetics

The initial MC on a wet basis of orange fruit was estimated under laboratory conditions using Eq. 1.

1 $$\:MC=\left[\frac{{W}_{w}-\:{W}_{d}}{{W}_{w}}\right]\times\:100$$

where $\:{W}_{w}\:and\:{W}_{d}\:$are the weight of fresh orange, g.

The DR is the speed at which moisture is removed from orange sample to reach equilibrium MC, typically measured in mass per unit area per time. It is crucial for optimizing drying processes, impacting energy use and product quality. And it was calculated using Eq. (2)^46^.

2 $$\:DR=\:\frac{{M}_{wt2}-{M}_{wt1}}{\varDelta\:t}$$

The weight loss of the henna sample (g) was calculated by subtracting the later weight ($\:{M}_{wt2}$) from the earlier weight ($\:{M}_{wt1}$), with $\:\varDelta\:t$ representing the time interval (h).

The MR represents the ratio of the MC at a given time ($\:{M}_{t}$) to the initial MC ($\:{M}_{0}$), making it a dimensionless value. The drying rate indicates the speed at which internal moisture is transferred to the surrounding environment. And it was determined using Eq. (3)^47^.

3 $$\:MR=\frac{{M}_{t}}{{M}_{0}}$$

### Mathematical modeling

Table 3 presents the mathematical models utilized in this study to analyze the thin-layer drying behavior of orange slices placed at various tray positions within an indirect mixed-mode solar dryer. These models were used to describe and simulate the drying kinetics by fitting them to the experimentally obtained MR data over time. They are categorized into two primary types: (1) semi-theoretical models, which are based on simplified derivations from Fick’s second law of diffusion and incorporate empirical constants to improve flexibility and accuracy; and (2) empirical models, which are purely based on statistical fitting of experimental data without direct physical interpretation. This classification allows for a more comprehensive understanding of the drying process, supporting the identification of the most appropriate model to describe the moisture removal characteristics of orange slices under the given solar drying conditions.

**Table 3:** Mathematical models applied to MR curves.

No.	Model name	Model	Category	Ref.
1	Aghbashlo	$\:MR=\mathrm{exp}\left(-\frac{{k}_{1}t}{1+{k}_{2}t}\right)$	Semi-Theoretical	^48,49^
2	Henderson - Pabis	$\:MR=a\:\mathrm{e}\mathrm{x}\mathrm{p}\left(-kt\right)$		^50–52^
3	Lewis (Newton)	$\:\mathrm{M}\mathrm{R}=\mathrm{e}\mathrm{x}\mathrm{p}\left(-\mathrm{k}\mathrm{t}\right)$		
4	Midilli	$\:\mathrm{M}\mathrm{R}=\mathrm{a}\mathrm{*}\mathrm{e}\mathrm{x}\mathrm{p}\left(-\mathrm{k}{\mathrm{t}}^{n}\right)+bt$		
5	Modified Midilli (I)	$\:\mathrm{M}\mathrm{R}=\mathrm{e}\mathrm{x}\mathrm{p}\left(-\mathrm{k}{\mathrm{t}}^{n}\right)+bt$		^53,54^
6	Modified Midilli (II)	$\:\mathrm{M}\mathrm{R}=\mathrm{a}\mathrm{*}\mathrm{e}\mathrm{x}\mathrm{p}\left(-\mathrm{k}{\mathrm{t}}^{n}\right)+b$		
7	Logarithmic (Asymptotic)	$\:\mathrm{M}\mathrm{R}=\mathrm{a}\mathrm{*}\mathrm{e}\mathrm{x}\mathrm{p}\left(-\mathrm{k}\mathrm{t}\right)+c$	Empirical	^50–52^
8	Modified Page	$\:\mathrm{M}\mathrm{R}=\mathrm{e}\mathrm{x}\mathrm{p}\left(-{\left(\mathrm{k}\mathrm{t}\right)}^{\mathrm{n}}\right)$		
9	Page	$\:\mathrm{M}\mathrm{R}=\mathrm{e}\mathrm{x}\mathrm{p}\left(-\mathrm{k}{\mathrm{t}}^{\mathrm{n}}\right)$		
10	Wang-Sigh	$\:MR=1+bt+a{t}^{2}$		
11	Weibullian (I)	$\:\mathrm{M}\mathrm{R}=\mathrm{e}\mathrm{x}\mathrm{p}\left(-{\left(\frac{t}{\alpha\:}\right)}^{\beta\:}\right)$		^53,54^
12	Weibullian (II)	$\:\mathrm{M}\mathrm{R}={10}^{-{\left(\frac{t}{\delta\:}\right)}^{n}}$		

### Effective moisture diffusivity ($\:{D}_{eff}$)

The $\:{D}_{eff}$ of orange slices was determined using the analytical solution of Fick’s second law of diffusion. This approach assumes several ideal conditions: a constant moisture diffusivity throughout the drying process, an infinite slab geometry representing the shape of the orange slices, and a uniform initial distribution of moisture within the sample. These assumptions simplify the mathematical modeling and allow for a more accurate estimation of how moisture moves from the interior of the slices to their surface during drying. The $\:{D}_{eff}$ (m^2^/s) was estimated using Eq. (4)^55^.

4 $$\:MR=\:\frac{8}{{\pi\:}^{2}}\times\:\sum_{n=1}^{\infty}\frac{1}{{(2n+1)}^{2}}exp\left(-\frac{{(2n+1)}^{2}\times\:{\pi\:}^{2}\times\:{D}_{eff}\times\:t}{4{L}^{2}}\right)$$

where, *L* is the half-thickness of the orange slice, m, and n is a positive integer, also called the Fourier’s series number. Simplified by taking the first term of the series solution:

5 $$\:MR=\:\frac{8}{{\pi\:}^{2}}\times\:exp\left(-\frac{{\pi\:}^{2}\times\:{D}_{eff}\times\:t}{4{L}^{2}}\right)$$

The $\:{D}_{eff}$ for drying can be calculated from the slope obtained by plotting the $\:{ln}\left(MR\right)$ against the drying time.

6 $$\:{ln}\left(MR\right)={ln}\left(\frac{8}{{\pi\:}^{2}}\right)-\left(\frac{{\pi\:}^{2}\times\:{D}_{eff}\times\:t}{4{L}^{2}}\right)$$

The $\:{D}_{eff}$ is also typically calculated by using the slope of Eq. (6), namely when the $\:{ln}\left(MR\right)$ versus time is plotted, a straight line with a slope *K* is obtained (Eq. 7). Then the $\:{D}_{eff}$ was estimated using Eq. 8.

7 $$\:k=\frac{{\pi\:}^{2}\times\:{D}_{eff}}{4{L}^{2}}$$

8 $$\:{D}_{eff}=\frac{4{L}^{2}\times\:k}{{\pi\:}^{2}}$$

### Statistical analysis

To evaluate the goodness of fit for the drying curves of the twelve mathematical models examined, several statistical parameters were employed—primarily the coefficient of determination (R²), the adjusted coefficient of determination ($\:{R}_{adj.}^{2}$), and the root mean square error (RMSE). Among these, R²_adj served as the principal criterion for identifying the most appropriate model to describe the drying behavior of orange slices in the solar dryer, as highlighted by^21^. Additional statistical metrics, such as RMSE, were also used to assess the accuracy of each model in predicting MC relative to experimental values. An ideal model is typically characterized by R² and $\:{R}_{adj.}^{2}$ values approaching 1.0, and RMSE values approaching zero, indicating a strong correlation and minimal prediction error, respectively^19,20^. The mathematical expressions for these statistical parameters are provided in Eqs. 8–10.

8 $$\:{R}^{2}=1-\frac{\sum\:_{i=1}^{N}{{(MR}_{pre,\:i}-{MR}_{obs,\:i})}^{2}}{\sum\:_{i=1}^{N}{{(\stackrel{-}{M}R}_{pre}-{MR}_{obs,\:i})}^{2}}$$

9 $$\:{R}_{adj.}^{2}=1-\left(1-{R}^{2}\right)*\frac{N-1}{N-n}$$

10 $$\:RMSE=\sqrt{\frac{1}{N}{\sum\:}_{i=1}^{N}{{(MR}_{pre,\:i}-{MR}_{obs,\:i})}^{2}}$$

where: $\:{MR}_{obs,\:\:i}$ and $\:{MR}_{pre,\:i}$are the *i*^*th*^ experimental and predicted values; $\:{\stackrel{-}{M}R}_{pre}\:$is the average predicted values; *N* is the number of observations; *n* is the number of constants in a model.

To determine the most appropriate mathematical model for accurately describing the thin-layer drying behavior of orange samples, twelve widely recognized non-linear regression models—outlined in Table 3—were systematically evaluated. These models were selected based on their frequent application in drying kinetics studies and their ability to capture the moisture removal dynamics of biological materials. The estimation of model parameters was conducted using non-linear regression analysis in Microsoft Excel Office 365 (version 15), which enabled precise curve fitting between the experimental MR data and the predicted values generated by each model. This approach facilitated a comprehensive comparison of model performance in terms of statistical accuracy and reliability.

Additionally, Residuals are a fundamental concept in model evaluation and play a crucial role in assessing the performance and validity of mathematical models—especially in drying kinetics and regression analysis. Where the residuals are the differences between the observed experimental values and the predicted values from the mathematical model (Eq. 11).

11 $$\:Residual={MR}_{obs,\:i}-{MR}_{pre,\:i}$$

### Environmental and economic analysis

Among the key considerations in adopting solar dryers for agricultural and industrial applications are their environmental and economic impacts. Enviro-economic analysis provides a comprehensive assessment that combines both environmental benefits—such as reduced greenhouse gas emissions and lower fossil fuel dependency—and economic performance indicators like energy cost savings and operational efficiency. One crucial metric in this context is the payback period, which refers to the time required to recover the initial investment through cost savings generated by the system. Evaluating the enviro-economic performance of a solar dryer involves analyzing energy consumption, drying efficiency, carbon footprint reduction, system costs, and potential economic returns. A shorter payback period, coupled with low environmental impact, enhances the feasibility and attractiveness of solar drying technologies for farmers, food processors, and policymakers.

The energy payback period (EPP) refers to the time required to recover the amount of energy consumed in producing the raw materials used to construct the IMMSD^56^. It is expressed in years and calculated using the following Eq.

12 $$\:EPP=\frac{{E}_{em}}{{E}_{at}}$$

where, $\:{E}_{em}$ is the embodied energy (kW.h) used for constructing IMMSD (Table 4) and $\:{E}_{at}$ annual thermal energy output, kWh/Yr.

**Table 4:** $\:{\boldsymbol{E}}_{\boldsymbol{e}\boldsymbol{m}}$ Of the materials used for construction Of the IMMSD^57–59^.

No.	Component	Material	$\:{\boldsymbol{E}}_{\boldsymbol{e}\boldsymbol{m}}$, kWh/kg	Quantity, kg	Total $\:{\boldsymbol{E}}_{\boldsymbol{e}\boldsymbol{m}}$, kWh
1	Metal frame	Metal	8.89	50	444.5
2	Glass cover	Glass	7.28	10	72.8
3	Insulation	Wood dust	2.0	4.0	8.0
4	Coating	Paint	25.11	2.0	50.22
5	Absorber plate	Galvanized iron sheet	9.636	10.5	101.178
6	Hinges	Metal	8.89	0.1	0.889
7	Handel	Metal	8.89	0.1	0.889
8	Drying trays	Metal	8.89	8.0	71.12
9	PV system	--	--	--	734.89
10	Battery	--	--	--	46.00
11	Battery charger	--	--	--	33.00
12	Air circulation fan				
	i) Copper wire	Copper	19.61	0.2	3.922
	ii) Casings, fan, shaft etc.	Steel	8.89	0.2	1.778
Total $\:{\boldsymbol{E}}_{\boldsymbol{e}\boldsymbol{m}}$, kWh					1569.186

The $\:{E}_{at}$ can be calculated using Eq. 13,

13 $$\:{E}_{at}={E}_{dt}\times\:{t}_{d}$$

where, $\:{E}_{dt}$ is the daily thermal energy output, kWh/day, and $\:{t}_{d}$ is the average number of sunlight days in a year, and it was about 365 day per year in Aswan city, Egypt.

14 $$\:{E}_{dt}=\frac{{M}_{es}\times\:{h}_{e}}{3.6\times\:{10}^{6}}$$

where, $\:{M}_{es}$ is the evaporative moisture from orange slices, kg, and $\:{h}_{e}$ is the latent heat for evaporation, J/kg.

The environmental impact of the IMMSD is assessed by estimating the average carbon dioxide (CO₂) emissions. For electricity generated from coal, approximately 0.98 kg of CO₂ is emitted per kilowatt-hour (kWh). Assuming a lifespan ($\:{L}_{\mathrm{I}\mathrm{M}\mathrm{M}\mathrm{S}\mathrm{D}}$) of 25 years for the IMMSD, the annual carbon dioxide emissions ($\:{CO}_{2,\:EY}$) can be calculated using Eq. (15)^56^.

15 $$\:{CO}_{2,\:EY}=\frac{{E}_{em}\times\:0.98\:kg}{{L}_{\mathrm{I}\mathrm{M}\mathrm{M}\mathrm{S}\mathrm{D}}}$$

Taking into account transmission losses ($\:{L}_{t}$) and domestic losses ($\:{L}_{d}$), the equation for $\:{CO}_{2,\:EY}$ is modified as shown in Eq. (16).

16 $$\:{CO}_{2,\:EY}=\frac{{E}_{em}\times\:0.98\:kg}{{L}_{\mathrm{I}\mathrm{M}\mathrm{M}\mathrm{S}\mathrm{D}}}\times\:\frac{1}{1-{L}_{t}}\times\:\frac{1}{1-{L}_{d}}$$

In the older machine, $\:{L}_{t}\:$and $\:{L}_{d}$ are assumed as 40% and 20%, respectively^56^.

The annual carbon dioxide reduction ($\:{CO}_{2,\:RA}$) achieved by the IMMSD can be calculated using a specific equation that quantifies the environmental benefit of replacing conventional energy sources with solar energy. This reduction represents the amount of CO₂ emissions that would have been produced if the same amount of energy had been generated using fossil fuels, such as coal. The $\:{CO}_{2,\:RA}$ can be determined using Eq. 17.

17 $$\:{CO}_{2,\:\:RA}=\left[{E}_{at}\times\:{L}_{\mathrm{I}\mathrm{M}\mathrm{M}\mathrm{S}\mathrm{D}}-{E}_{em}\right]\times\:2.042\:kg$$

The emission carbon credit (ECC) represents the avoidance of one metric ton (1,000 kg) of CO₂ emissions. The carbon credit earned from the operation of the developed IMMSD was calculated using Eq. (18)^60^.

18 $$\:ECC=\:Net\:mitigation\:of\:{CO}_{2}in\:life\:time\:\times\:Price\:per\:ton\:of\:{CO}_{2}\:mitigation\:$$

Economic analysis is essential for evaluating the IMMSD, as it helps determine if the system is financially viable, cost-effective, sustainable, and suitable for investment. Ane it was assessed using Eqs. 19–24. Table 5 presents the main assumptions applied in the analysis, reflecting the economic conditions in Egypt.

**Table 5:** Calculation assumptions of economic analysis of the IMMSD.

Parameter	Nomenclature	Unit	Value
Capital cost	$\:{C}_{CC}$	USD	700
Interest rate	$\:d$	%	27.25%
Maintenance cost	$\:{C}_{m}$	USD/year	3% of the $\:{C}_{ac}$
Salvage value	$\:{V}_{a}$	%	8% of the $\:{C}_{ac}$
Operating life	$\:{L}_{\mathrm{I}\mathrm{M}\mathrm{M}\mathrm{S}\mathrm{D}}$	year	25
Inflation rate	$\:i$	%	18
Number of operating days per year	--	days	90
Cost of fresh orange	--	USD/kg	0.5
Cost of dried slice orange	--	USD/kg	5

The annualized investment cost ($\:{C}_{a}$) of the IMMSD was calculated using parameters in Eq. (19)^61,62^.

19 $$\:{C}_{a}={C}_{ac}+{C}_{m}-{V}_{a}$$

Where the $\:{C}_{ac}$ was estimated according to Eq. 20.

20 $$\:{C}_{ac}={C}_{cc}\times\:{F}_{c}$$

21 $$\:{F}_{c}=\:\frac{{d(1+d)}^{{L}_{\mathrm{I}\mathrm{M}\mathrm{M}\mathrm{S}\mathrm{D}}}}{{(1+d)}^{{L}_{\mathrm{I}\mathrm{M}\mathrm{M}\mathrm{S}\mathrm{D}}}-1}$$

where $\:{C}_{cc}$ is the total capital cost, USD, $\:{F}_{c}$ is the capital recovery factor, $\:d$ is the interest rate, %.

The drying cost per one kilogram ($\:{C}_{s}$) is calculated using Eq. 22.

22 $$\:{C}_{ds}={C}_{fd}\times\:\frac{{M}_{f}}{{M}_{d}}+\frac{{C}_{a}\times\:{D}_{d}}{{M}_{d}\times\:D\:}$$

where, $\:{C}_{dp}$ is the drying cost of in USD/kg, $\:{C}_{fd}$ is the cost of fresh orange in USD/kg, $\:{M}_{f\:}and\:{M}_{d}$ is the quantity of fresh and dried orange in kg, *D* is the number of operating days per year, $\:{D}_{d}$ is the drying days per batch.

The savings obtained from the IMMSD after a (j) number of years are given by Eq. 23.

23 $$\:{S}_{j}=\frac{\left[{S}_{pc}-{C}_{ds}\right]\times\:{M}_{d}}{D}\times\:D\times\:{\left(1+j\right)}^{j-1}$$

where $\:{S}_{pc}$ is the selling price of dried orange slices in USD/kg.

The investigation payback period (IPP) for the IMMSD is calculated using Eq. (24)^63^.

24 $$\:\mathrm{I}\mathrm{P}\mathrm{P}=\frac{ln\left[1-\frac{{C}_{cc}}{{S}_{1}}(d-i)\right]}{\mathrm{ln}\left(\frac{1+i}{1+d}\right)}$$

where, $\:{S}_{1}$ is the cost savings after the first year, USD/year.

### Uncertainty analysis

The measurement uncertainties for temperature, relative humidity, wind speed, and solar radiation were found to be 0.34%, 0.29%, 0.23%, and 0.14%, respectively. Taking all these variables into account, the overall uncertainty in evaluating the efficiency of the SD was estimated at around ± 2%.

25 $$\:{\mathcal{W}}_{r}={\left[{\left(\frac{\partial\:R}{\partial\:{x}_{1}}{\mathcal{W}}_{1}\right)}^{2}+{\left(\frac{\partial\:R}{\partial\:{x}_{2}}{\mathcal{W}}_{2}\right)}^{2}+\dots\:+{\left(\frac{\partial\:R}{\partial\:{x}_{3}}{\mathcal{W}}_{3}\right)}^{2}\right]}^{1/2}$$

## Results and discussions

### Drying conditions

**Fig. 3:**
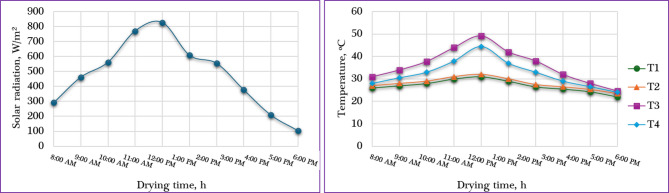
Average solar radiation and temperature during the experiment period. Whereas (T1) is the ambient air temperature, (T2) is the inlet air temperature to the solar collector, (T3) is the outlet air temperature from the solar collector/inlet air temperature to the drying room, and (T4) is the outlet air temperature to the drying room.

The drying experiments for fresh orange slices were carried out in January 2025 at Aswan University, Egypt. The study investigated the effects of three slice thicknesses—4 mm, 6 mm, and 8 mm—as well as the vertical position of the drying trays within the solar dryer (lower, middle, and upper levels) on the drying performance. Each treatment was tested in triplicate to ensure consistency, and the laboratory analyses revealed an average initial MC of 85.6% (wet basis) for the fresh orange slices. During the experimental period, the ambient environmental conditions varied, with air temperatures in the shade ranging from 22 °C to 32 °C, solar radiation intensities between 88 and 826 W/m², and natural wind speeds fluctuating from 0.1 to 0.4 m/s. Inside the IMMSD, the drying air temperature ranged from 24.6 °C to 49.2 °C, reflecting the effect of solar heating and airflow control within the system. Figure 3 illustrates the average solar radiation intensity, along with the temperature profiles inside and outside the IMMSD throughout the drying period, providing insight into the thermal behavior of the system. All drying experiments were conducted under a constant air velocity of 1 m/s, ensuring consistent airflow conditions for comparative analysis across all treatments. This controlled setup allowed for the accurate assessment of how slice thickness and tray position affect the drying kinetics of orange slices.

### Drying kinetics

**Fig. 4:**
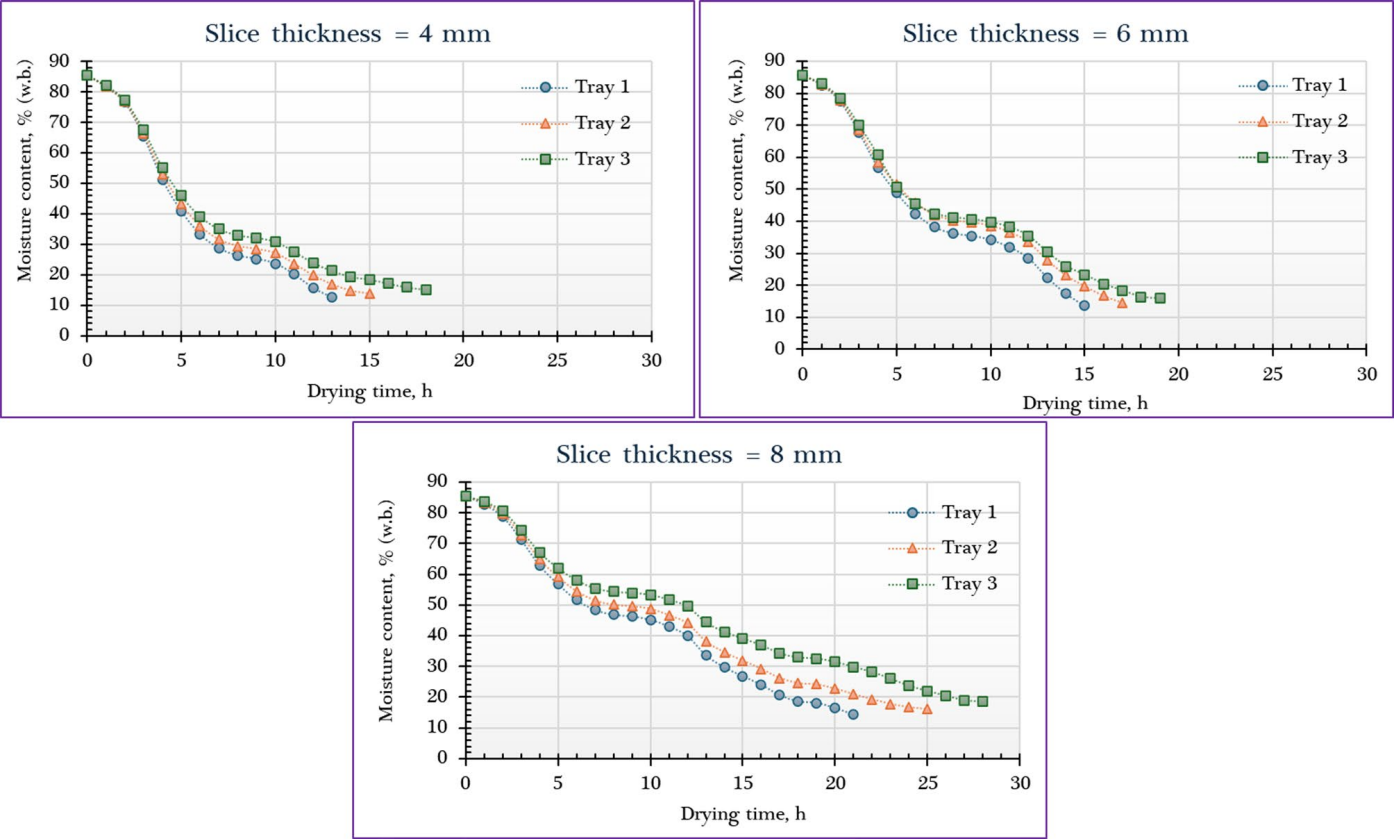
MC of orange dried using IMMSD at different slice thicknesses and tray positions.

Figure 4 illustrates the variation in MC of orange slices dried using the IMMSD across different slice thicknesses and tray positions. The data reveal that the MR decreased exponentially with increasing drying time, a behavior characteristic of falling-rate drying periods, with no observable constant-rate phase. This drying pattern aligns with previous findings in similar studies involving herbs and food materials, where an initial rapid moisture loss—due to surface water evaporation—is followed by a gradual decline as internal moisture diffusion becomes the rate-limiting step. This continuous decrease in MR over time strongly suggests that internal diffusion was the dominant mechanism governing mass transfer throughout the drying process. The initial weights of the orange slices varied according to slice thickness and tray position. For the 4 mm slices, the initial weights were 1890 g, 1920 g, and 1910 g on the lower, middle, and upper trays, respectively. At 6 mm thickness, the corresponding weights were 2815 g, 2820 g, and 2810 g. For the thickest slices, 8 mm, the initial weights measured 3800 g on the lower tray, 2820 g on the middle tray, and 3985 g on the upper tray. These variations reflect both the slice thickness and the packing or distribution on each tray, which can influence the drying behavior and efficiency in the IMMSD system.

As shown in Fig. 4, the initial MC of the fresh orange slices was measured at 85.6% ± 0.16% (w.b.), while the required time to reach the final MC range varied between 13 and 25 h, depending on the slice thickness and tray position. Shorter drying durations were observed for thinner slices (4 mm) and lower trays (about 13 h), while the longest drying times were recorded for thicker slices (8 mm) and upper trays (about 25 h). This is expected, as increasing the slice thickness extends the moisture diffusion path, thereby slowing down the drying process and resulting in higher final MCs. At the lower tray, where air is hottest, the lowest final MC was about 12.5% for 4 mm slices, compared to 13.6% and 14.4% for slices of 6 mm and 8 mm, respectively. Conversely, reducing slice thickness accelerated moisture removal, led to shorter drying times, and resulted in lower final MC. These findings are consistent with previously reported studies^64–71^. Additionally, many previous studies showed that in multi-tray or multi-zone dryers, trays located in zones with higher temperatures or better airflow (often closer to the air inlet or heat source) dry faster and reach lower final MC than those in less favorable positions^68,69,72^.

**Fig. 5:**
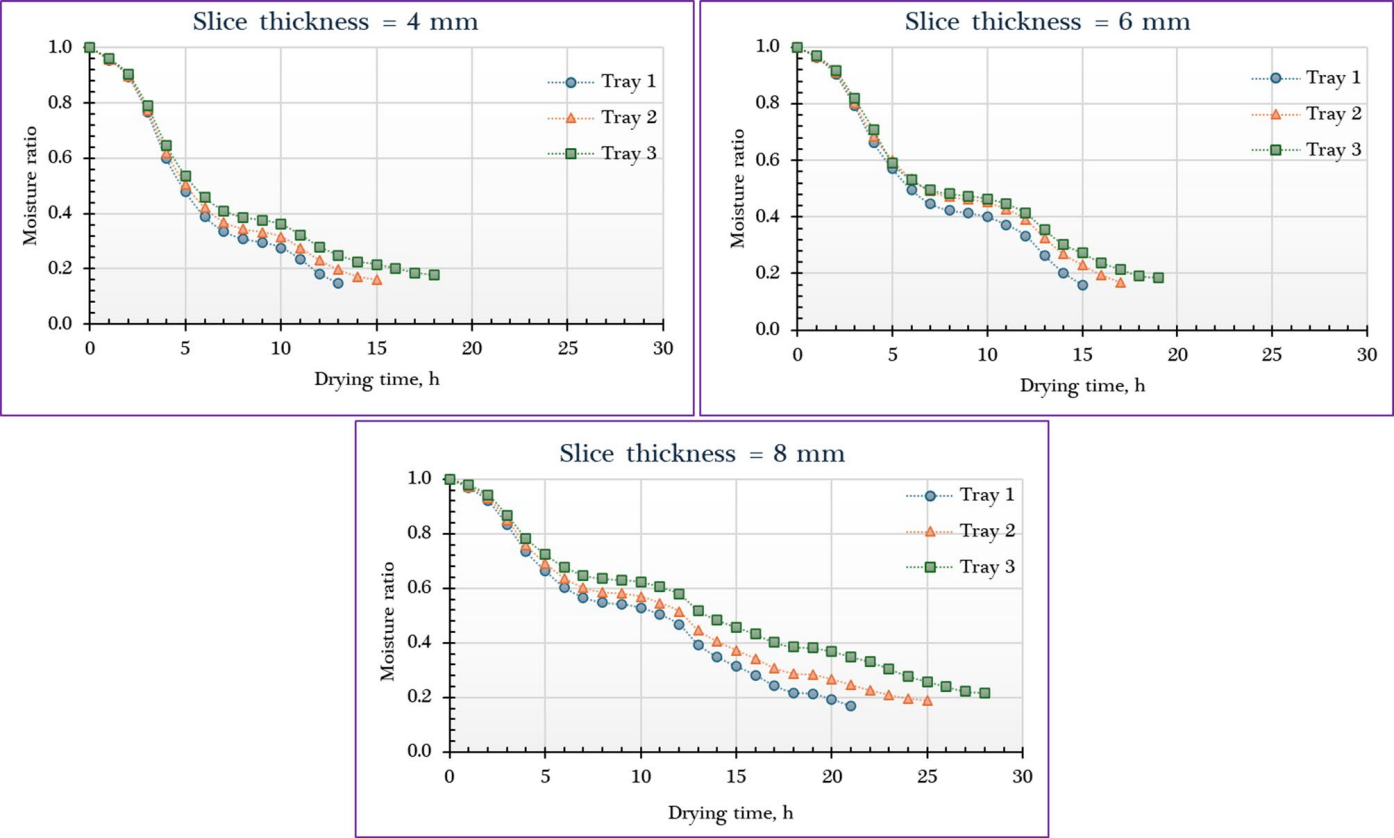
Moisture ratio of orange dried using IMMSD at different slice thicknesses and tray positions.

Figure 5 presents the relationship between the MR and drying time for orange slices dried in the IMMSD under varying slice thicknesses and tray positions. At the beginning of the drying process, MR values were relatively high for all slice thicknesses. As solar radiation levels increased throughout the day—from 88 to 826 W/m²—the MR progressively declined. This reduction was more pronounced due to the high initial MC of the orange slices and the efficient heat absorption by the IMMSD’s collector plate, which enhanced the drying rate. However, as solar radiation decreased in the later hours, the drying rate slowed, requiring more time to remove the remaining MC. The drying behavior of high-moisture agricultural products like orange typically follows two main stages. In the initial phase, drying occurs rapidly due to the abundant surface moisture, with a significant portion of thermal energy being used to evaporate this free water. Simultaneously, some of the heat penetrates the product, increasing its internal temperature and preparing it for the second phase. During this phase, capillary forces play a critical role in transporting internal moisture toward the surface, enabling internal mass transfer, while external mass transfer continues to remove moisture from the surface. This combined mechanism supports efficient drying during the early period, particularly under strong solar radiation and optimal system performance.

Slice thickness and tray position are both critical factors influencing the final MR—the proportion of moisture remaining in a product after the drying process. Thicker slices tend to retain more moisture due to the longer diffusion path required for internal moisture to reach the surface, which slows down the drying process. Numerous studies on products such as potato, onion puree, and rough rice have demonstrated that thinner layers (e.g., 2–2.5 cm) result in significantly lower final MRs than thicker ones (e.g., 6–10 cm). For instance, 2.5 mm potato slices achieved a final MR as low as 0.001, while 5 mm slices had higher ratios ranging from 0.003 to 0.650^65,67,73,74^.

In addition to layer thickness, the position of trays within a dryer also impacts drying efficiency. Trays in less favorable positions—such as the middle or upper levels, or those farther from the primary heat source or airflow—often exhibit higher final MRs, indicating less effective drying. Conversely, trays in more favorable positions, such as the lower levels or those exposed to stronger airflow and higher temperatures, typically achieve lower final MCs. For example, in a heat pump dryer, the lowest tray yielded a lower final MC compared to the highest tray. This spatial variation can lead to non-uniform drying, ultimately affecting product quality unless tray positions and airflow are carefully optimized^69,72^.

**Fig. 6:**
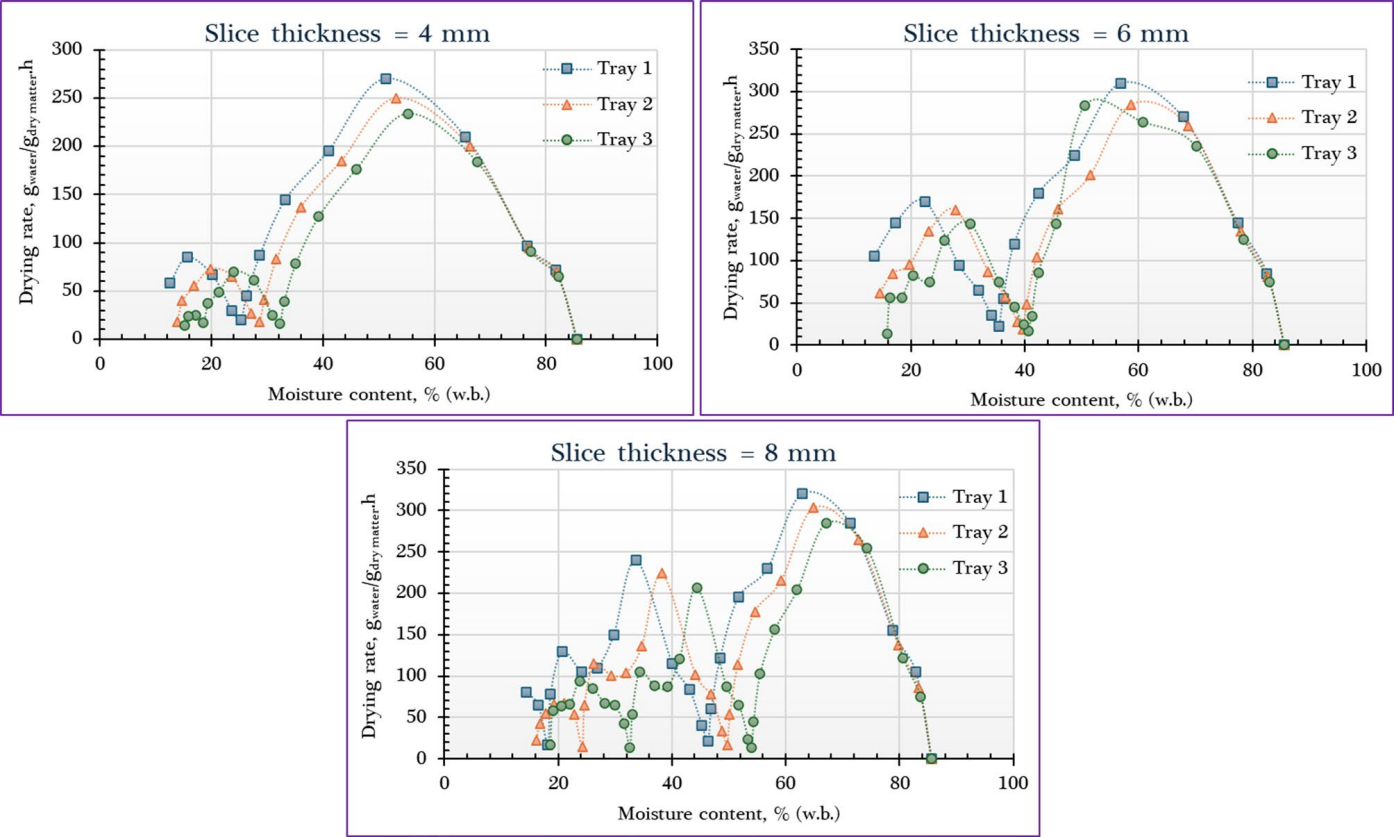
Relationship between the drying rate and the corresponding moisture content of orange dried using IMMSD at different slice thicknesses and tray positions.

Figure 6 illustrates the relationship between drying rate and corresponding moisture content for orange slices dried using the IMMSD across various slice thicknesses and tray positions. The figure highlights how both physical and positional factors influence the rate at which moisture is removed during the drying process. The data clearly demonstrate that the highest DR occurred in orange slices placed on the lower trays compared to those on the middle and upper trays. This is primarily attributed to the higher temperatures experienced at the lower level due to direct exposure to the incoming hot air from the solar collector. For instance, at a slice thickness of 4 mm, the DR was approximately 270, 250, and 234 g _water_/g _dry matter_/h for the lower, middle, and upper trays, respectively. In terms of slice thickness, the drying rate increased with greater thickness, reaching about 270, 310, and 320 g _water_/g _dry matter_/h for 4, 6, and 8 mm slices, respectively at the lower trays. This increase in DR with slice thickness is likely due to the higher loading capacity and greater total moisture content, which enhanced the moisture evaporation rate during the initial stages of drying. Overall, both tray position and slice thickness had a significant impact on the drying rate, with optimal conditions found in thicker slices placed on lower trays.

These results are consistent with findings reported in earlier studies. For example, Elshehawy et al. ^75^ confirmed that elevated air temperatures enhance moisture diffusion from the interior to the surface of agricultural products, thereby accelerating surface evaporation and increasing the overall drying rate. In a related study, Darvishi et al. ^76^ found that the DR consistently declines as the MC of the product decreases during drying. Their observations revealed that the maximum drying rate occurs at the beginning of the process, during which free moisture is rapidly removed, especially in the case of high-moisture materials like orange slices. As drying progresses, the rate gradually decreases due to the reduced availability of free moisture and increasing internal resistance to moisture movement. For all tested slice thicknesses in the current study, drying predominantly followed a falling-rate period, with only a short-lived constant-rate phase at the very beginning. This drying behavior suggests that internal moisture diffusion is the controlling mechanism, emphasizing that the resistance within the product—rather than surface evaporation—plays the primary role in determining total drying time. Furthermore, Elshehawy et al. ^75^ also noted that the initial reduction in MR tends to occur more slowly compared to the later stages of drying, supporting the idea that internal resistance becomes more influential as surface moisture is depleted.

**Fig. 7:**
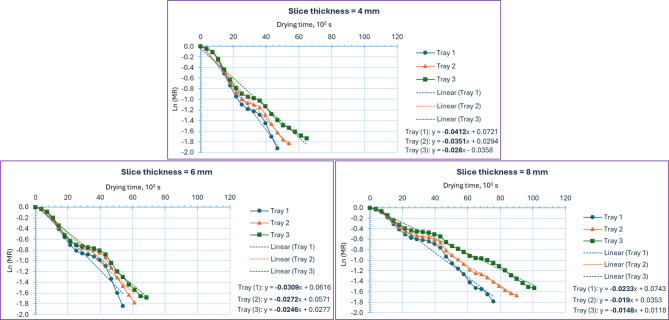
Relationship between Ln(MR) vs. drying time of orange dried using IMMSD at different slice thicknesses and tray positions.

Figure 7 presents the relationship between the natural logarithm of the moisture ratio [Ln(MR)] and the drying time for orange slices dried using the IMMSD under various experimental conditions. Specifically, the graph highlights how this correlation varies with changes in slice thickness (4, 6, and 8 mm) and the position of the drying trays (lower, middle, and upper levels). This figure is essential as it provides the graphical representation needed to determine the drying rate constant, denoted as k. By analyzing the slope of the linear portion of the Ln(MR) versus drying time curve, the value of k can be accurately extracted. The drying constant k is a critical parameter in drying kinetics, as it reflects the rate at which moisture is removed from the product. Once the value of k is obtained from the experimental data, it can be further used to estimate the D_eff_ of the orange slices by applying Eq. 8. Where the drying constant is a key parameter in drying kinetics models, reflecting how quickly moisture is removed from food slices during drying. Both slice thickness and drying temperature have significant and opposite effects on the drying constant^16,19,20^.

The equations presented in Fig. 7 clearly demonstrate that increasing the slice thickness leads to a reduction in the drying constant (*k*), indicating a slower moisture removal rate with thicker slices. Specifically, the drying constant decreased from 0.0412 s⁻¹ to 0.0309 s⁻¹ as the orange slice thickness increased from 4 mm to 6 mm, and further declined to 0.0233 s⁻¹ when the thickness was increased to 8 mm. This inverse relationship between slice thickness and drying constant highlights the increased internal resistance to moisture diffusion in thicker slices, which prolongs the drying process. Where thicker slices have a longer path for moisture to travel from the interior to the surface, slowing down the drying rate and resulting in a lower drying constant. This trend is observed in sweet potato, Hami melon, crabapple, and apple slices, where thinner slices dry faster and have higher drying constants^77–80^.

Additionally, tray position played a significant role in influencing the value of the drying constant (*k*). The lower tray, which is directly exposed to the incoming hot air from the solar collector—typically the hottest and most energy-rich air—exhibited the highest drying constant compared to the middle and upper trays. This indicates a more efficient drying process at the lower level due to the higher thermal energy available. At a slice thickness of 4 mm, the drying constants were 0.0412 s⁻¹, 0.0351 s⁻¹, and 0.0280 s⁻¹ for the lower, middle, and upper trays, respectively. This trend underscores the effect of temperature gradients within the drying chamber, where reduced heat intensity at higher tray levels results in slower drying rates and lower *k* values. Where higher temperatures provide more energy for moisture evaporation, accelerating the drying process and raising the drying constant. This effect is consistent across various fruits and vegetables, including sweet potato, Hami melon, crabapple, pumpkin, and apple^77–82^.

**Fig. 8:**
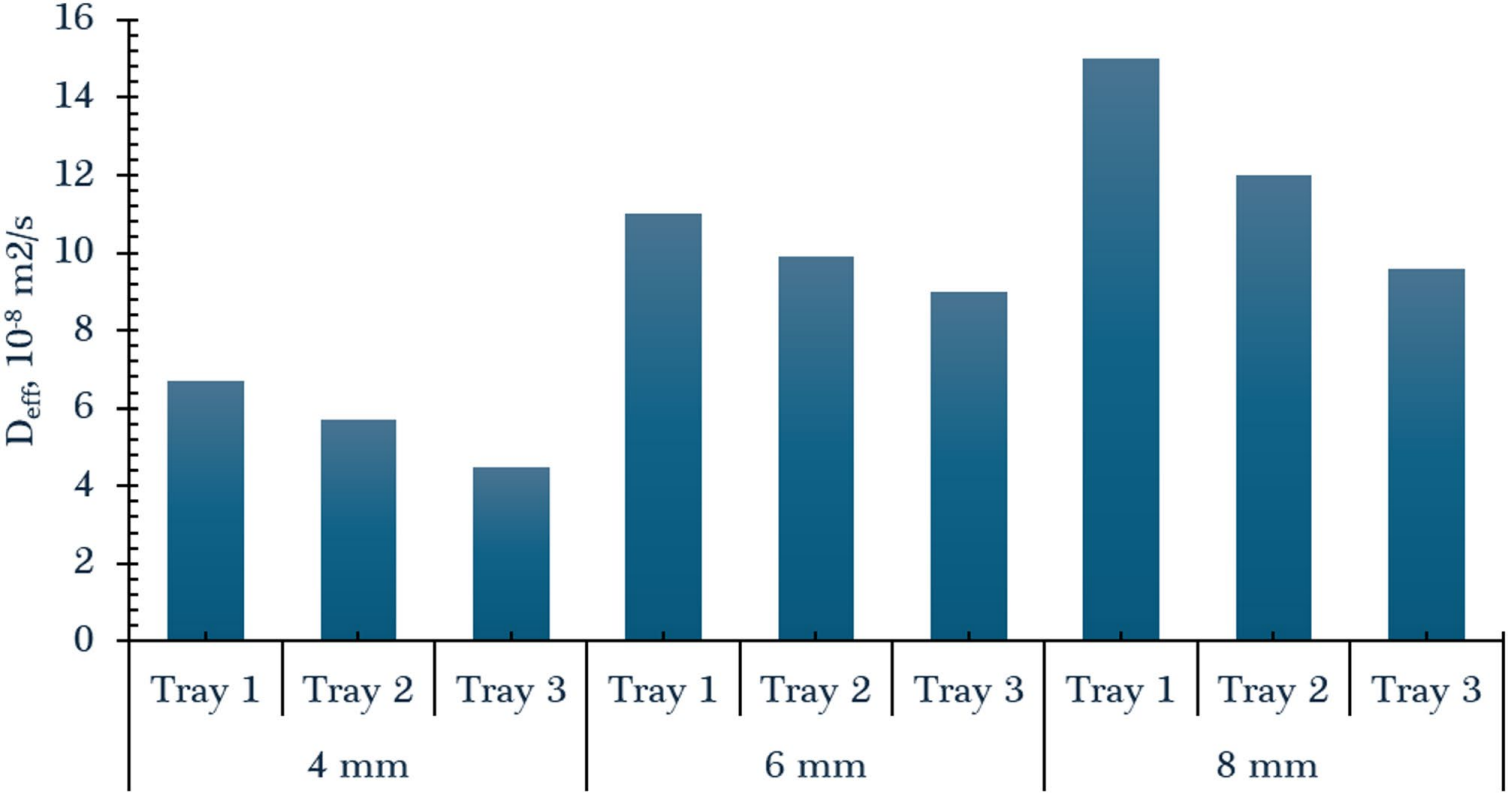
D_eff_ of orange dried using IMMSD at different slice thicknesses and tray positions.

On the other hand, the D_eff_ is a critical parameter that quantifies the ease with which moisture migrates from the interior of the product to its surface during the drying process. It serves as a fundamental indicator in the mathematical modeling of drying behavior and is essential for the optimization, control, and scale-up of solar drying systems. A higher Deff value signifies more efficient internal moisture transport, which translates to faster drying rates under given conditions. As illustrated in Fig. 8, the D_eff_ values showed a noticeable increase with increasing slice thickness. Specifically, when the orange slice thickness was raised from 4 mm to 8 mm, the effective diffusivity increased from 6.7 × 10⁻⁸ m²/s to 15 × 10⁻⁸ m²/s. This trend suggests that thicker slices may retain higher internal moisture gradients, which can enhance the driving force for diffusion, particularly in the initial drying stages. It also implies that while thicker slices slow surface evaporation, they may promote internal moisture movement under sufficient thermal energy. Most studies report that increasing slice thickness leads to higher effective moisture diffusivity values, even though thicker slices require longer drying times. This trend is observed in products like tomato, sweet potato, potato, peach, and kiwi, where thicker slices facilitate greater internal moisture movement, resulting in higher diffusivity coefficients. However, thinner slices dry faster due to shorter moisture paths, but their effective diffusivity is often lower^83–88^.

Furthermore, the tray position, the specific drying zone within the dryer—has a substantial impact on the D_eff_ of orange slices. This variation is primarily attributed to the temperature gradient that exists within the dryer. Orange slices placed on the lower tray, which is directly exposed to the hottest air exiting the solar collector, exhibited the highest diffusivity values. This is because higher temperatures enhance the mobility of water molecules, thereby accelerating the internal moisture diffusion process. At a slice thickness of 4 mm, the effective diffusivity was approximately 6.7 × 10⁻⁸ m²/s on the lower tray, compared to 5.7 × 10⁻⁸ m²/s on the middle tray, and 4.5 × 10⁻⁸ m²/s on the upper tray. These results clearly demonstrate that moisture removal efficiency decreases with tray height, reflecting the reduced thermal energy available at higher levels of the drying chamber. Such findings underscore the importance of airflow and temperature distribution in optimizing drying performance across different zones of the dryer. This is attributed to more favorable thermal conditions and air movement, which enhance moisture removal^79,89^. Finally, increasing slice thickness generally increases effective moisture diffusivity, while optimal tray position (exposure to higher temperature/airflow) further enhances diffusivity and reduces drying time. Both factors are crucial for efficient drying and product quality. Table 6 shows the comparison between the obtained D_eff_ with previous studies related to orange drying.

**Table 6:** Comparison between the obtained D_eff_ with previous studies related to orange drying.

Material	Drying Method	Temperature, °C	Slice Thickness	D_eff_ (m²/s)	Notes/Findings	Ref.
Orange slice	Convective	40–80	2, 4, and 6 mm	0.627–3.50 × 10⁻⁹	D_eff_ increases with temperature and air velocity; thickness has little effect	^90,91^
Orange slice	Infrared	Not specified	6 mm	1.59–2.49 × 10⁻¹⁰	D_eff_ increases with IR power	^92^
Orange pomace	Convective	50, 60, 70	Not specified	0.334–1.06 × 10⁻⁹	D_eff_ increases with temperature	^93^
Orange peel	Convective	40, 50, 60	3 mm	0.086–2.017 × 10⁻⁸	D_eff_ increases with temperature and blanching temperature	^94^
Orange powder	Microwave	Not specified	Not specified	1.69–5.00 × 10⁻¹⁰	D_eff_ increases with microwave power	^95^
Orange slice	**IMMSD**	**Max. 49.2**	**4**,** 6**,** and 8 mm**	**4.5 to 15 × 10** ^**–8**^	**D**_**eff**_ **peaked in lower trays and with thicker orange slices.**	**Current study**

### Mathematical modeling

Table 7 presents the statistical evaluation results—specifically the coefficient of determination (R²), adjusted R^2^, R^2^_adj_., and RMSE —which were used to assess how well each of the twelve examined drying models fit the experimental drying data, as supported by references. The selection of the most appropriate model was primarily based on achieving R^2^, and R^2^_adj_.values as close as possible to 1.0, indicating strong predictive accuracy, along with RMSE values near zero, which reflect minimal prediction error. Based on these criteria, five semi-theoretical models—Midilli, Modified Midilli (I), Modified Midilli (II), Aghbashlo, and Henderson–Pabis—emerged as the most suitable for accurately describing the thermal behavior illustrated in the drying curves obtained from the IMMSD. Their performance varied according to both the drying tray position and the thickness of the orange slices. For the lower tray (Tray 1), Modified Midilli (I) and Modified Midilli (II) provided the best fit for orange slices of 4 mm and 8 mm thickness, respectively, while the Henderson–Pabis model was most appropriate for slices with a 6 mm thickness. On the middle tray (Tray 2), Modified Midilli (II), Aghbashlo, and Henderson–Pabis models best described the drying behavior of orange slices with thicknesses of 4 mm, 6 mm, and 8 mm, respectively. As for the upper tray (Tray 3), the Modified Midilli (II) model offered the best fit for 4 mm slices, Henderson–Pabis for 6 mm, and Modified Midilli (I) for 8 mm thickness.

Collectively, these findings highlight the Midilli, Modified Midilli (I), Modified Midilli (II), Aghbashlo, and Henderson–Pabis models as the most accurate and reliable in predicting the drying kinetics of orange slices in the IMMSD system. These models showed strong correlation between the predicted and experimental moisture ratios, confirming their validity for this application. However, it is important to note that while these models effectively describe the moisture content evolution over time, they do not account for the interactive effects of other drying variables, such as air velocity and drying temperature. The scope of the present study was limited to a constant air velocity of 1 m/s and drying temperatures that did not exceed 49.2 °C. Therefore, future studies should consider a broader range of operational parameters to further validate and refine these models.

**Fig. 9:**
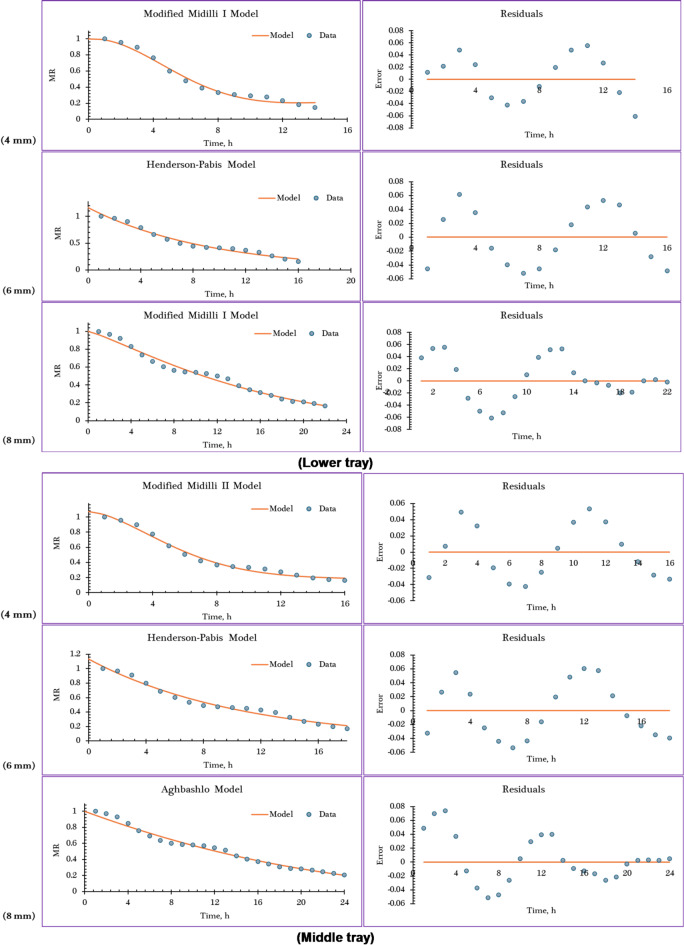

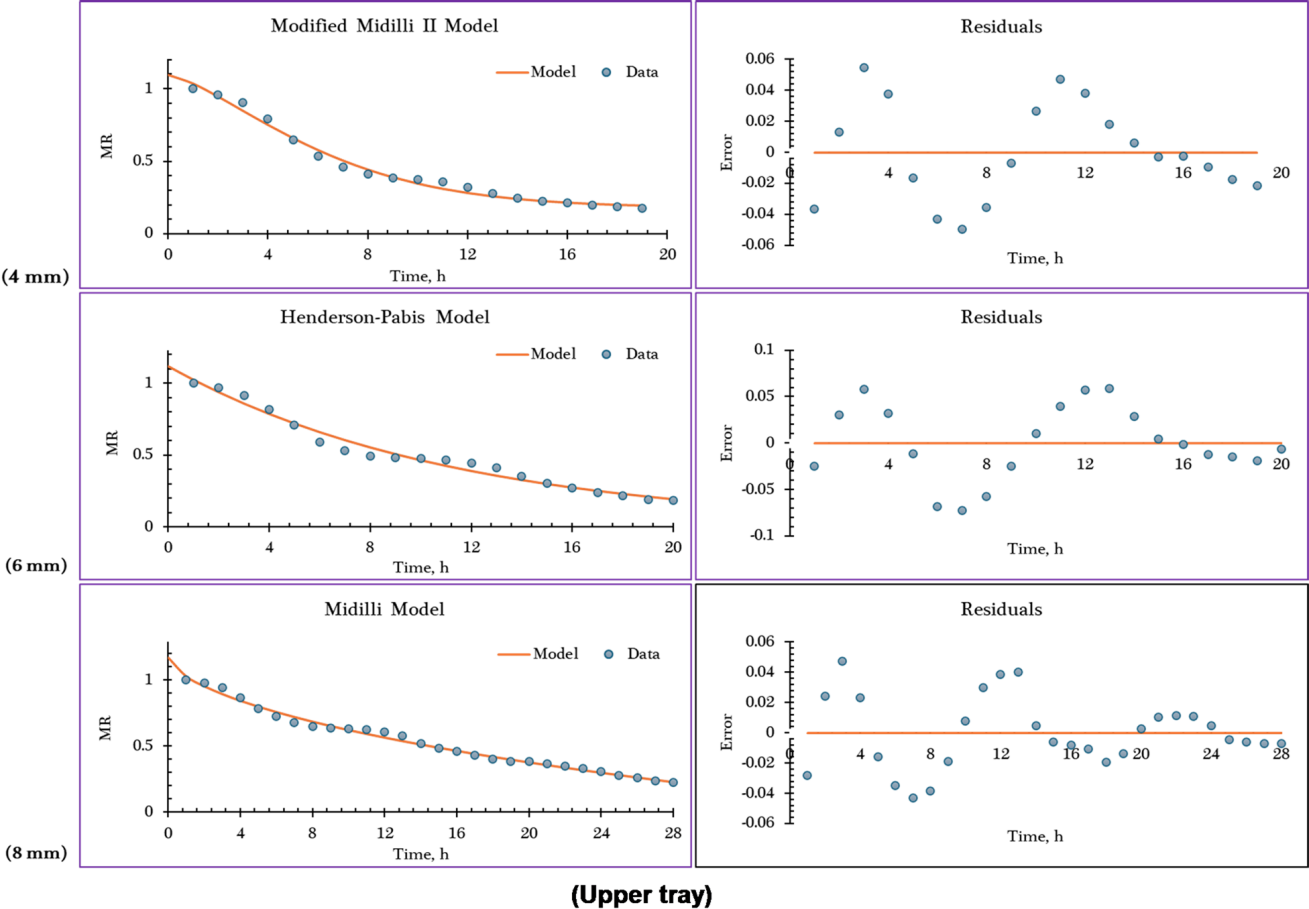
Observed and predicted MR, along with the corresponding residuals, over drying time for the best-fitting models at various slice thicknesses and tray positions.

Table 7 presents the constants of the mathematical models and their goodness-of-fit parameters at different slice thicknesses and drying tray positions. And Fig. 9 illustrates the observed and predicted MR, along with the corresponding residuals, over drying time for the best-fitting models at various slice thicknesses and tray positions.

**Table 7:** Constants of the mathematical models and their goodness-of-fit parameters at different slice thicknesses and drying tray positions.

Thin layer models	Layer thickness, mm		Models’ constants values										Goodness of fit indices		
			Constants			Values		Standard Error		*p*-value*			RMSE	*R* ^2^	*R*^2^_adj_.
Tray no. 1 (Lower tray)															
Aghbashlo	**4**		**k**_**1**_			0.1146		0.0138		**2.65 × 10** ^**− 6**^			0.0549	0.9688	0.9662
			**K**_**2**_			-0.0195		0.0117		0.1218					
	**6**		**k**_**1**_			0.0932		0.0093		**9.17 × 10** ^**− 8**^			0.0451	0.9733	0.9714
			**K**_**2**_			-0.0088		0.0089		0.3388					
	**8**		**k**_**1**_			0.0651		0.0039		**3.63 × 10** ^**− 13**^			0.0330	0.9846	0.9838
			**K**_**2**_			-0.0101		0.0036		**0.0120**					
Henderson - Pabis	**4**		**k**			0.1478		0.0083		**5.02 × 10** ^**− 10**^			0.0499	0.9743	0.9721
			**a**			1.0806		0.0349		**8.07 × 10** ^**− 13**^					
	**6**		**k**			0.1078		0.0051		**4.78 × 10** ^**− 12**^			0.0424	0.9764	0.9748
			**a**			1.0458		0.0271		**1.25 × 10** ^**− 15**^					
	**8**		**k**			0.0781		0.0027		**7.06 × 10** ^**− 18**^			0.0352	0.9824	0.9815
			**a**			1.0319		0.0197		**7.16 × 10** ^**− 23**^					
Lewis (Newton)	**4**		**k**			0.1349		0.0068		**4.44 × 10** ^**− 11**^			0.0579	0.9624	0.9624
	**6**		**k**			0.1017		0.0037		**3.48 × 10** ^**− 14**^			0.0450	0.9716	0.9716
	**8**		**k**			0.0750		0.0019		**3.38 × 10** ^**− 21**^			0.0365	0.9801	0.9801
Logarithmic (Asymptotic)	**4**		**k**			0.1371		0.0296		**0.0007**			0.0517	0.9746	0.9700
			**a**			1.1131		0.0980		**2.05 × 10** ^**− 7**^					
			**c**			-0.0395		0.1117		**0.7300**					
	**6**		**k**			0.1049		0.0224		**0.0004**			0.0440	0.9765	0.9729
			**a**			1.0582		0.0998		**9.07 × 10** ^**− 8**^					
			**c**			-0.0148		0.1133		0.8980					
	**8**		**k**			0.0576		0.0103		**2.07 × 10** ^**− 7**^			0.0329	0.9853	0.9838
			**a**			1.1925		0.1112		**1.69 × 10** ^**− 9**^					
			**c**			-0.1844		0.1225		0.1487					
Midilli	**4**		**k**			0.0730		0.0199		**0.0043**			0.0371	0.9881	0.9845
			**a**			1.0195		0.0323		**2.41 × 10** ^**− 11**^					
			**b**			0.0125		0.0032		**0.0028**					
			**n**			1.4996		0.1618		**3.17 × 10** ^**− 6**^					
	**6**		**k**			0.0934		0.0298		**0.0086**			0.0452	0.9771	0.9713
			**a**			1.0319		0.0420		**1.25 × 10** ^**− 11**^					
			**b**			0.0028		0.0075		0.7123					
			**n**			1.0951		0.2022		**0.0002**					
	**8**		**k**			0.0778		0.0214		**0.0019**			0.0330	0.9861	0.9838
			**a**			1.0246		0.0309		**1.36 × 10** ^**− 17**^					
			**b**			-0.0080		0.0062		0.2120					
			**n**			0.8792		0.1695		**6.21 × 10** ^**− 5**^					
Modified Midilli (I)	**4**		**k**			0.0650		0.0122		**0.0002**			0.0361	0.9876	0.9854
			**b**			0.0130		0.0028		**0.0008**					
			**n**			1.5588		0.1250		**7.81 × 10** ^**− 8**^					
	**6**		**k**			0.0783		0.0160		**0.0003**			0.0446	0.9757	0.9720
			**b**			0.0042		0.0066		0.5412					
			**n**			1.1769		0.1578		**4.77 × 10** ^**− 6**^					
	**8**		**k**			0.0653		0.0099		**2.48 × 10** ^**− 6**^			0.0327	0.9855	0.9840
			**b**			-0.0067		0.0054		0.2354					
			**n**			0.9538		0.1271		**4.28 × 10** ^**− 7**^					
Modified Midilli (II)	**4**		**k**			0.0640		0.0209		**0.0119**			0.0343	0.9899	0.9868
			**a**			0.8214		0.0438		**4.02 × 10** ^**− 9**^					
			**b**			0.1932		0.0264		**2.55 × 10** ^**− 5**^					
			**n**			1.6848		0.2023		**8.28 × 10** ^**− 6**^					
	**6**		**k**			0.0958		0.0294		**0.0069**			0.0449	0.9774	0.9717
			**a**			0.9302		0.1611		**8.81 × 10** ^**− 5**^					
			**b**			0.1000		0.1410		0.4916					
			**n**			1.1379		0.2301		**0.0003**					
	**8**		**k**			0.0571		0.0166		**0.0030**			0.0331	0.9860	0.9836
			**a**			1.5360		0.7239		**0.0480**					
			**b**			-0.5115		0.7090		0.4799					
			**n**			0.8760		0.1603		**3.44 × 10** ^**− 5**^					
Modified Page	**4**		**k**			0.1369		0.0048		**2.38 × 10** ^**− 12**^			0.0473	0.9768	0.9749
			**n**			1.2303		0.0949		**2.04 × 10** ^**− 8**^					
	**6**		**k**			0.1031		0.0035		**5.29 × 10** ^**− 14**^			0.0436	0.9751	0.9733
			**n**			1.1019		0.0782		**1.16 × 10** ^**− 9**^					
	**8**		**k**			0.0758		0.0017		**1.65 × 10** ^**− 21**^			0.0341	0.9835	0.9827
			**n**			1.1005		0.0515		**3.03 × 10** ^**− 15**^					
Page	**4**		**k**			0.0866		0.0166		**0.0002**			0.0473	0.9768	0.9749
			**n**			1.2303		0.0949		**2.04 × 10** ^**− 8**^					
	**6**		**k**			0.0818		0.0141		**4.56 × 10** ^**− 5**^			0.0436	0.9751	0.9733
			**n**			1.1019		0.0782		**1.16 × 10** ^**− 9**^					
	**8**		**k**			0.0585		0.0076		**2.02 × 10** ^**− 7**^			0.0341	0.9835	0.9827
			**n**			1.1005		0.0515		**3.03 × 10** ^**− 15**^					
Wang-Sigh	**4**		**b**			-0.1145		0.0074		**2.88 × 10** ^**− 9**^			0.0530	0.9709	0.9685
			**a**			0.0038		0.0007		**0.0002**					
	**6**		**b**			-0.0875		0.0053		**1.29 × 10** ^**− 10**^			0.0462	0.9720	0.9700
			**a**			0.0023		0.0004		**0.0001**					
	**8**		**b**			-0.0621		0.0024		**5.15 × 10** ^**− 17**^			0.0338	0.9838	0.9829
			**a**			0.0011		0.0001		**2.13 × 10** ^**− 7**^					
Weibullian (I)	**4**		**β**			1.2303		0.0949		**2.04 × 10** ^**− 8**^			0.0473	0.9768	0.9749
			**α**			7.3060		0.2584		**2.38 × 10** ^**− 12**^					
	**6**		**β**			1.1019		0.0782		**1.16 × 10** ^**− 9**^			0.0436	0.9751	0.9733
			**α**			9.6968		0.3288		**5.29 × 10** ^**− 14**^					
	**8**		**β**			1.1005		0.0515		**3.03 × 10** ^**− 15**^			0.0341	0.9835	0.9827
			**α**			13.1896		0.2953		**1.65 × 10** ^**− 21**^					
Weibullian (II)	**4**		**n**			1.2303		0.0949		**2.04 × 10** ^**− 8**^			0.0473	0.9768	0.9749
			**δ**			14.3914		0.9296		**2.71 × 10** ^**− 9**^					
	**6**		**n**			1.1019		0.0782		**1.16 × 10** ^**− 9**^			0.0436	0.9751	0.9733
			**δ**			20.6702		1.4533		**1.03 × 10** ^**− 9**^					
	**8**		**n**			1.1005		0.0515		**3.03 × 10** ^**− 15**^			0.0341	0.9835	0.9827
			**δ**			28.1434		1.2941		**2.17 × 10** ^**− 15**^					
Tray no. 2 (Middel tray)															
Aghbashlo		**4**		**k**_**1**_	0.1140		0.0117			**1.27 × 10** ^**− 7**^		0.0488		0.9732	0.9713
				**K**_**2**_	-0.0080		0.0098			0.4290					
		**6**		**k**_**1**_	0.0836		0.0073			**4.02 × 10** ^**− 9**^		0.0417		0.9757	0.9742
				**K**_**2**_	-0.0066		0.0070			0.3587					
		**8**		**k**_**1**_	0.0606		0.0029			**6.51 × 10** ^**− 17**^		0.0280		0.9881	0.9877
				**K**_**2**_	-0.0044		0.0027			0.1182					
Henderson - Pabis		**4**		**k**	0.1316		0.0061			**3.83 × 10** ^**− 12**^		0.0432		0.9790	0.9775
				**a**	1.0622		0.0289			**2.54 × 10** ^**− 15**^					
		**6**		**k**	0.0939		0.0041			**1.06 × 10** ^**− 13**^		0.0405		0.9770	0.9756
				**a**	1.0326		0.0246			**8.34 × 10** ^**− 18**^					
		**8**		**k**	0.0666		0.0017			**3.07 × 10** ^**− 23**^		0.0282		0.9880	0.9875
				**a**	1.0208		0.0148			**3.85 × 10** ^**− 29**^					
Lewis (Newton)		**4**		**k**	0.1225		0.0048			**1.00 × 10** ^**− 13**^		0.0482		0.9719	0.9719
		**6**		**k**	0.0900		0.0029			**1.70 × 10** ^**− 16**^		0.041		0.9745	0.9745
		**8**		**k**	0.0649		0.0012			**1.46 × 10** ^**− 27**^		0.0287		0.9870	0.9870
Logarithmic (Asymptotic)		**4**		**k**	0.1406		0.0221			**2.45 × 10** ^**− 5**^		0.0445		0.9793	0.9761
				**a**	1.0384		0.0595			**2.09 × 10** ^**− 10**^					
				**c**	0.0309		0.0676			0.6553					
		**6**		**k**	0.0869		0.0181			**0.0002**		0.0417		0.9772	0.9742
				**a**	1.0681		0.1019			**2.67 × 10** ^**− 8**^					
				**c**	-0.0419		0.1152			0.7212					
		**8**		**k**	0.0600		0.0071			**1.75 × 10** ^**− 8**^		0.0282		0.9885	0.9875
				**a**	1.0679		0.0569			**1.94 × 10** ^**− 15**^					
				**c**	-0.0564		0.0651			0.3949					
Midilli		**4**		**k**	0.0854		0.0215			**0.0019**		0.0371		0.9867	0.9834
				**a**	1.0271		0.0329			**7.30 × 10** ^**− 13**^					
				**b**	0.0097		0.0029			**0.0058**					
				**n**	1.3270		0.1426			**7.72 × 10** ^**− 7**^					
		**6**		**k**	0.0956		0.0298			**0.0064**		0.0428		0.9775	0.9727
				**a**	1.0323		0.0406			**4.00 × 10** ^**− 13**^					
				**b**	-0.0037		0.0084			0.6689					
				**n**	0.9437		0.2036			**0.0004**					
		**8**		**k**	0.0709		0.0164			**0.0003**		0.0285		0.9887	0.9872
				**a**	1.0225		0.0258			**5.69 × 10** ^**− 22**^					
				**b**	-0.0028		0.0032			0.3812					
				**n**	0.9305		0.1190			**8.62 × 10** ^**− 8**^					
Modified Midilli I		**4**		**k**	0.0737		0.0129			**7.29 × 10** ^**− 5**^		0.0367		0.9859	0.9837
				**b**	0.0103		0.0026			**0.0017**					
				**n**	1.3960		0.1127			**1.42 × 10** ^**− 8**^					
		**6**		**k**	0.0793		0.0150			**9.11 × 10** ^**− 5**^		0.0425		0.9763	0.9731
				**b**	-0.0024		0.0076			0.7573					
				**n**	1.0234		0.1608			**1.27 × 10** ^**− 5**^					
		**8**		**k**	0.0601		0.0078			**8.39 × 10** ^**− 8**^		0.0285		0.9883	0.9873
				**b**	-0.0019		0.0027			0.5013					
				**n**	0.9978		0.0859			**4.18 × 10** ^**− 11**^					
Modified Midilli II		**4**		**k**	0.0816		0.0236			**0.0047**		0.0350		0.9882	0.9852
				**a**	0.8419		0.0485			**7.19 × 10** ^**− 10**^					
				**b**	0.1812		0.0301			**5.97 × 10** ^**− 5**^					
				**n**	1.4536		0.1734			**2.32 × 10** ^**− 6**^					
		**6**		**k**	0.0882		0.0216			**0.0011**		0.0430		0.9774	0.9725
				**a**	1.1399		0.3726			**0.0085**					
				**b**	-0.1084		0.3544			0.7641					
				**n**	0.9529		0.2112			**0.0005**					
		**8**		**k**	0.0641		0.0102			**2.55 × 10** ^**− 6**^		0.0286		0.9887	0.9872
				**a**	1.1778		0.2362			**5.44 × 10** ^**− 5**^					
				**b**	-0.1554		0.2229			0.4930					
				**n**	0.9262		0.1229			**1.56 × 10** ^**− 7**^					
Modified Page		**4**		**k**	0.1233		0.0042			**4.79 × 10** ^**− 14**^		0.0450		0.9771	0.9755
				**n**	1.1276		0.0783			**8.77 × 10** ^**− 10**^					
		**6**		**k**	0.0908		0.0028			**6.17 × 10** ^**− 16**^		0.0413		0.9761	0.9746
				**n**	1.0668		0.0683			**4.20 × 10** ^**− 11**^					
		**8**		**k**	0.0652		0.0011			**4.33 × 10** ^**− 27**^		0.0282		0.9880	0.9875
				**n**	1.0507		0.0376			**8.07 × 10** ^**− 20**^					
Page		**4**		**k**	0.0944		0.0158			**3.35 × 10** ^**− 5**^		0.0450		0.9771	0.9755
				**n**	1.1276		0.0783			**8.77 × 10** ^**− 10**^					
		**6**		**k**	0.0773		0.0123			**1.03 × 10** ^**− 5**^		0.0413		0.9761	0.9746
				**n**	1.0668		0.0683			**4.20 × 10** ^**− 11**^					
		**8**		**k**	0.0568		0.0057			**5.31 × 10** ^**− 10**^		0.0282		0.9880	0.9875
				**n**	1.0507		0.0376			**8.07 × 10** ^**− 20**^					
Wang-Sigh		**4**		**b**	-0.1068		0.0053			**9.06 × 10** ^**− 12**^		0.0463		0.9758	0.9741
				**a**	0.0035		0.0004			**1.70 × 10** ^**− 6**^					
		**6**		**b**	-0.0769		0.0041			**3.11 × 10** ^**− 12**^		0.0437		0.9732	0.9716
				**a**	0.0018		0.0003			**3.03 × 10** ^**− 5**^					
		**8**		**b**	-0.0561		0.0016			**2.60 × 10** ^**− 22**^		0.0292		0.9872	0.9866
				**a**	0.0010		7.96E-05			**1.35 × 10** ^**− 11**^					
Weibullian (I)		**4**		**β**	1.1276		0.0783			**8.77 × 10** ^**− 10**^		0.0450		0.9771	0.9755
				**α**	8.1109		0.2731			**4.79 × 10** ^**− 14**^					
		**6**		**β**	1.0668		0.0683			**4.20 × 10** ^**− 11**^		0.0413		0.9761	0.9746
				**α**	11.0145		0.3441			**6.17 × 10** ^**− 16**^					
		**8**		**β**	1.0506		0.0376			**8.07 × 10** ^**− 20**^		0.0282		0.9880	0.9875
				**α**	15.3284		0.2701			**4.33 × 10** ^**− 27**^					
Weibullian (II)		**4**		**n**	1.1276		0.0783			**8.77 × 10** ^**− 10**^		0.0450		0.9771	0.9755
				**δ**	16.9937		1.0591			**2.08 × 10** ^**− 10**^					
		**6**		**n**	1.0668		0.0683			**4.20 × 10** ^**− 11**^		0.0413		0.9761	0.9746
				**δ**	24.0717		1.5808			**6.10 × 10** ^**− 11**^					
		**8**		**n**	1.0506		0.0376			**8.07 × 10** ^**− 20**^		0.0282		0.9880	0.9875
				**δ**	33.9041		1.2352			**1.23 × 10** ^**− 19**^					
Tray no. 3 (Upper tray)															
Aghbashlo	**4**			**k**_**1**_	0.1126		0.0097		**1.64 × 10** ^**− 9**^		0.0431			0.9765	0.9751
				**k**_**1**_	0.0050		0.0082		0.5472						
	**6**			**k**_**1**_	0.0841		0.0070		5.08 × 10^− 10^		0.0419			0.9751	0.9737
				**k**_**1**_	-0.0012		0.0066		0.8529						
	**8**			**k**_**1**_	0.0519		0.0023		**4.74 × 10** ^**− 19**^		0.0258			0.9882	0.9877
				**k**_**1**_	-0.0004		0.0024		0.8721						
Henderson - Pabis	**4**			**k**	0.1118		0.0047		**1.87 × 10** ^**− 14**^		0.0415			0.9783	0.9770
				**a**	1.0359		0.0261		**3.29 × 10** ^**− 18**^						
	**6**			**k**	0.0881		0.0037		**3.90 × 10** ^**− 15**^		0.0407			0.9764	0.9751
				**a**	1.0248		0.0239		**1.43 × 10** ^**− 19**^						
	**8**			**k**	0.0524		0.0012		**2.29 × 10** ^**− 26**^		0.0259			0.9881	0.9877
				**a**	1.0016		0.0125		**1.21 × 10** ^**− 33**^						
Lewis (Newton)	**4**			**k**	0.1071		0.0034		**3.82 × 10** ^**− 17**^		0.0425			0.9759	0.9759
	**6**			**k**	0.0853		0.0025		**2.06 × 10** ^**− 18**^		0.0408			0.9750	0.9750
	**8**			**k**	0.0523		0.0008		**4.05 × 10** ^**− 32**^		0.0254			0.9881	0.9881
Logarithmic (Asymptotic)	**4**			**k**	0.1424		0.0157		**1.05 × 10** ^**− 7**^		0.0376			0.9832	0.9811
				**a**	0.9669		0.0360		**9.87 × 10** ^**− 15**^						
				**c**	0.0990		0.0383		**0.0200**						
	**6**			**k**	0.0933		0.0158		**1.76 × 10** ^**− 5**^		0.0418			0.9766	0.9738
				**a**	1.0019		0.0681		**4.23 × 10** ^**− 11**^						
				**c**	0.0285		0.0789		0.7224						
	**8**			**k**	0.0498		0.0060		**9.17 × 10** ^**− 9**^		0.0263			0.9882	0.9873
				**a**	1.0252		0.0584		**6.27 × 10** ^**− 16**^						
				**c**	-0.0275		0.0662		0.6807						
Midilli	**4**			**k**	0.0937		0.0204		**0.0004**		0.0342			0.9869	0.9843
				**a**	1.0325		0.0305		**1.42 × 10** ^**− 15**^						
				**b**	0.0082		0.0021		**0.0015**						
				**n**	1.2008		0.1144		**2.63 × 10** ^**− 8**^						
	**6**			**k**	0.0974		0.0293		**0.0043**		0.0430			0.9767	0.9723
				**a**	1.0354		0.0402		**1.89 × 10** ^**− 14**^						
				**b**	-0.0007		0.0058		0.9026						
				**n**	0.9524		0.1747		**5.34 × 10** ^**− 5**^						
	**8**			**k**	0.0717		0.0157		**0.0001**		0.0250			0.9898	0.9885
				**a**	1.0248		0.0231		**2.69 × 10** ^**− 25**^						
				**b**	-0.0046		0.0031		0.1495						
				**n**	0.8155		0.1152		**2.01 × 10** ^**− 7**^						
Modified Midilli (I)	**4**			**k**	0.0794		0.0121		**6.65 × 10** ^**− 6**^		0.0346			0.9858	0.9840
				**b**	0.0087		0.0019		**0.0004**						
				**n**	1.2721		0.0920		**2.57 × 10** ^**− 10**^						
	**6**			**k**	0.0799		0.0151		**5.86 × 10** ^**− 5**^		0.0429			0.9753	0.9724
				**b**	0.0004		0.0052		0.9445						
				**n**	1.0334		0.1380		**8.86 × 10** ^**− 7**^						
	**8**			**k**	0.0590		0.0070		**6.23 × 10** ^**− 9**^		0.0252			0.9892	0.9884
				**b**	-0.0036		0.0028		0.2094						
				**n**	0.8908		0.0849		**7.75 × 10** ^**− 11**^						
Modified Midilli (II)	**4**			**k**	0.0934		0.0227		**0.0009**		0.0323			0.9884	0.9860
				**a**	0.8443		0.0442		**6.10 × 10** ^**− 12**^						
				**b**	0.1843		0.0264		**4.48 × 10** ^**− 6**^						
				**n**	1.3052		0.1373		**9.67 × 10** ^**− 8**^						
	**6**			**k**	0.0957		0.0233		**0.0008**		0.0430			0.9766	0.9722
				**a**	1.0354		0.2221		**0.0003**						
				**b**	-0.0011		0.2031		0.9959						
				**n**	0.9685		0.1888		**0.0001**						
	**8**			**k**	0.0534		0.0112		**7.09 × 10 − 5**		0.0251			0.9897	0.9884
				**a**	1.4592		0.5168		**0.0092**						
				**b**	-0.4346		0.5049		0.3976						
				**n**	0.8145		0.1126		**1.40 × 10** ^**− 7**^						
Modified Page	**4**			**k**	0.1072		0.0035		**2.30 × 10** ^**− 16**^		0.0436			0.9760	0.9746
				**n**	1.0164		0.0639		**1.20 × 10** ^**− 11**^						
	**6**			**k**	0.0856		0.0026		**1.57 × 10** ^**− 17**^		0.0417			0.9753	0.9739
				**n**	1.0254		0.0625		**2.83 × 10** ^**− 12**^						
	**8**			**k**	0.0522		0.0009		**3.95 × 10** ^**− 30**^		0.0258			0.9882	0.9877
				**n**	0.9928		0.0325		**1.74 × 10** ^**− 22**^						
Page	**4**			**k**	0.1033		0.0151		**2.80 × 10** ^**− 6**^		0.0436			0.9760	0.9746
				**n**	1.0164		0.0639		**1.20 × 10** ^**− 11**^						
	**6**			**k**	0.0804		0.0121		**2.99 × 10** ^**− 6**^		0.0417			0.9753	0.9739
				**n**	1.0254		0.0625		**2.83 × 10** ^**− 12**^						
	**8**			**k**	0.0533		0.0049		**1.86 × 10** ^**− 11**^		0.0258			0.9882	0.9877
				**n**	0.9928		0.0325		**1.74 × 10** ^**− 22**^						
Wang-Sigh	**4**			**b**	-0.0966		0.0036		**2.13 × 10** ^**− 15**^		0.0410			0.9788	0.9775
				**a**	0.0029		0.0002		**1.67 × 10** ^**− 9**^						
	**6**			**b**	-0.0748		0.0036		**5.22 × 10** ^**− 14**^		0.0447			0.9715	0.9700
				**a**	0.0017		0.0002		**1.01 × 10** ^**− 6**^						
	**8**			**b**	-0.0467		0.0013		**3.87 × 10** ^**− 24**^		0.0290			0.9851	0.9845
				**a**	0.0007		5.99E-05		**5.70 × 10** ^**− 12**^						
Weibullian (I)	**4**			**β**	1.0164		0.0639		**1.20 × 10** ^**− 11**^		0.0436			0.9760	0.9746
				**α**	9.3297		0.3025		**2.30 × 10** ^**− 16**^						
	**6**			**β**	1.0254		0.0625		**2.83 × 10** ^**− 12**^		0.0417			0.9753	0.9739
				**α**	11.6860		0.3553		**1.57 × 10** ^**− 17**^						
	**8**			**β**	0.9928		0.0325		**1.74 × 10** ^**− 22**^		0.0258			0.9882	0.9877
				**α**	19.1517		0.3232		**3.95 × 10** ^**− 30**^						
Weibullian (II)	**4**			**n**	1.0164		0.0639		**1.20 × 10** ^**− 11**^		0.0436			0.9760	0.9746
				**δ**	21.1960		1.2987		**8.06 × 10** ^**− 12**^						
	**6**			**n**	1.0254		0.0625		**2.83 × 10** ^**− 12**^		0.0417			0.9753	0.9739
				**δ**	26.3568		1.6709		**5.54 × 10** ^**− 12**^						
	**8**			**n**	0.9928		0.0325		**1.74 × 10** ^**− 22**^		0.0258			0.9882	0.9877
				**δ**	44.3668		1.6469		**4.77 × 10** ^**− 21**^						

* bold numbers were significant at *p* ≤ 0.05.

The validity, consistency, and robustness of the three selected drying models were confirmed by comparing the predicted MR values generated by the models with the actual experimental MR data, as illustrated in Fig. 9. The graphical representation revealed a strong agreement between the predicted and experimental data points, with the plotted values aligning closely along a 45° line. This indicates that the models exhibit a high degree of accuracy and reliability in capturing the drying kinetics of orange slices when processed using the IMMSD. Such alignment is a strong indicator of the models’ predictive capability and statistical adequacy. Furthermore, the near-linear relationship between predicted and observed values reinforces the applicability of these models in thin-layer drying simulations, demonstrating their effectiveness in describing the moisture removal behavior during the drying process. The empirical equations derived from these models are particularly useful for optimizing drying parameters and improving the energy efficiency of solar drying systems. Specifically, the drying behavior of orange slices under the tested conditions was best represented by the Midilli, Modified Midilli (I), Modified Midilli (II), Aghbashlo, and Henderson–Pabis models. These models have proven suitable for characterizing the drying dynamics, offering valuable tools for further analysis and design of efficient solar drying operations.

### Environmental analysis

Assessing the environmental impact of the IMMSD is essential for determining its sustainability and long-term advantages for drying orange slices. Table 8 outlines the $\:{E}_{em}$ of the materials used in constructing the IMMSD, which totaled approximately 1569.186 kWh. The associated lifetime CO₂ emissions were estimated at 128.05 metric tons, indicating the environmental and energy costs linked to the system’s production and installation. The analysis also shows that the energy required for moisture evaporation from orange slices ($\:{E}_{at}$) increased with increasing the slice thickness, amounting to roughly 916.56, 1372.31, and 1839.76 kWh for slices of 4, 6, and 8 mm, respectively. Notably, the 8 mm slices produced the highest annual energy output, largely due to their lower moisture content and quicker drying time. Consequently, the IMMSD achieved its greatest annual dried product yield when processing 8 mm slices, resulting in significant environmental and economic benefits. This setup also yielded the highest net CO₂ reduction over the system’s lifetime—90.72 tons—and shortened the energy payback period to only 0.85 years. Additionally, these environmental benefits translated into notable financial returns through carbon credits, with values of approximately 2179.29, 3342.61, and 4535.76 USD for slice thicknesses of 4, 6, and 8 mm, respectively.

**Table 8:** Environmental analysis of the IMMSD.

Parameters	Slice thickness		
	4 mm	6 mm	8 mm
Total moisture removed, g/day	4000.00	5989.00	8029.00
Daily energy output, W	2511.11	3759.76	5040.43
Annual thermal energy output ($\:{\boldsymbol{E}}_{\boldsymbol{a}\boldsymbol{t}}$), kW	916.56	1372.31	1839.76
Total embodied energy ($\:{\boldsymbol{E}}_{\boldsymbol{e}\boldsymbol{m}}$,), kWh	1569.186	1569.186	1569.186
EPP, years	1.71	1.14	0.85
Annual CO_2_ emission, ton	128.05	128.05	128.05
Annual CO_2_ reduction, ton	1.74	2.67	3.63
Net CO_2_ mitigation in lifetime, ton	43.59	66.85	90.72
Carbon credit earned (CCE), USD	2179.29	3342.61	4535.76

### Economic analysis

The main aim of the economic analysis in this study was to evaluate the feasibility and cost-effectiveness of operating the IMMSD for drying orange slices, with a focus on identifying the most economically advantageous slice thickness to maximize cost savings. The analysis employed Eqs. 19–24, which covered life cycle cost savings and the simple payback period method. Key economic parameters, outlined in Tables 8 and 9, were taken into account, including current economic conditions in Egypt in 2025 and projected costs of the IMMSD components. The overall capacity of the IMMSD was about (5.7 kg), (8.5 kg), and (11.5 kg). According to Table 9, the total capital investment required for the IMMSD was relatively low—approximately 700 USD—with an annualized capital cost of 143.75 USD. This helped keep the total annual operating cost of the system down to just 996.12 USD. The findings reveal that deploying the IMMSD for drying orange slices can lead to considerable yearly savings of 3220.5 USD, 6804.6 USD, and 14,015.9 USD for slice thicknesses of 4, 6, and 8 mm, respectively (Table 10). The payback period, which represents the time needed to recover the initial investment, was found to be exceptionally short—0.259, 0.123, and 0.059 years for slice thicknesses of 6 mm, 4 mm, and 8 mm, respectively. Compared to the IMMSD’s expected operational life of 25 years, this reflects a highly efficient and economically viable investment. In fact, under normal conditions and assuming no unforeseen issues, the initial capital cost of 700 USD could be recouped in about one month, underscoring the dryer’s strong cost-effectiveness and potential for rapid return on investment.

**Table 9:** Economical parameters related to the IMMSD.

Parameters	Value, USD
Capital cost	700
Annual capital cost	143.75
Annual maintenance cost	4.31
Annual salvage value	11.5
Annualized investment cost	136.56
Annual cost	996.12

**Table 10:** Economic analysis of the IMMSD.

Parameters	Slice thickness		
	4 mm	6 mm	8 mm
Dried quantity, kg/year	170	245.6	357.6
Saving after 1 year	3220.5	6804.6	14015.9
Investigation payback period, year	0.259	0.123	0.059

The annual quantity of dried oranges produced is influenced by only two primary factors: the dryer load capacity (determined by the three racks) and the total drying time. Both of these factors are directly dependent on the thickness of the orange slices. As discussed in the article, 4 mm slices have the shortest drying time; however, the thicker 8 mm slices allow for a significantly larger dryer load, which outweighs the advantage of the shorter drying duration. Consequently, from an economic and environmental standpoint, 8 mm slices offer greater efficiency due to higher throughput and lower relative energy consumption. Nevertheless, this does not automatically translate into superior dried product quality, as texture, color, and flavor can be affected by slice thickness.

The aim of examining the effect of shelf position within the drying chamber is not to assess economic or environmental feasibility, but rather to evaluate the uniformity of moisture content across different shelves and to determine the precise time at which each shelf reaches equilibrium moisture content. Understanding these variations enables more informed operational decisions, such as whether shelves should be periodically rotated during the drying process or if the chamber design should be modified to promote uniform airflow and temperature distribution. Such adjustments would enhance moisture homogeneity, improve overall product consistency, and potentially shorten the total drying time without compromising quality.

## Conclusions

The present study employed an IMMSD to dry orange slices under real climatic conditions at Aswan University, Egypt, during January 2025. The study aimed to evaluate the performance of the IMMSD by experimenting with different slice thicknesses (4, 6, and 8 mm) and varying the position of the drying trays within the drying chamber (lower, middle, and upper levels). A comprehensive comparative analysis was conducted to assess the impact of these variables on drying behavior. This included mathematical modeling of drying curves, drying kinetics, and determination of D_eff_, as well as an in-depth environmental and economic evaluation. The findings aim to bridge the existing research gap and contribute to the development of more efficient and standardized solar drying practices for citrus processing industries. The obtained results can be concluded as follows:

- The average initial moisture content of orange slices was about 85.6% (wet basis).
- The thinner orange slices (4 mm) placed on lower trays in the IMMSD dried faster and reached lower final moisture content due to shorter diffusion paths and higher tray temperatures.
- Thinner slices at lower trays dried faster and reached lower final moisture ratios than thicker ones due to shorter internal diffusion paths, confirming that both slice thickness and tray position critically influence drying efficiency.
- The thinner slices and lower trays achieving lower final moisture ratios due to improved heat transfer and shorter moisture diffusion paths.
- The D_eff_ values showed a noticeable increase with increasing slice thickness. Specifically, when the orange slice thickness was raised from 4 mm to 8 mm, the effective diffusivity increased from 6.7 × 10⁻⁸ m²/s to 15 × 10⁻⁸ m²/s.
- The tray position has a substantial impact on the D_eff_ of orange slices. This is because higher temperatures enhance the mobility of water molecules, thereby accelerating the internal moisture diffusion process. At a slice thickness of 4 mm, the effective diffusivity was approximately 6.7 × 10⁻⁸ m²/s on the lower tray, compared to 5.7 × 10⁻⁸ m²/s on the middle tray, and 4.5 × 10⁻⁸ m²/s on the upper tray.
- The findings of the mathematical modeling highlight the Midilli, Modified Midilli (I), Modified Midilli (II), Aghbashlo, and Henderson–Pabis models as the most accurate and reliable in predicting the drying kinetics of orange slices in the IMMSD system.
- The environmental analysis showed that the energy required for moisture evaporation increased with slice thickness, reaching 916.56, 1372.31, and 1839.76 kWh for 4, 6, and 8 mm slices, respectively. The 8 mm slices yielded the highest annual dried product output, net CO₂ reduction (90.72 tons), and the shortest energy payback time (0.85 years). Carbon credits ranged from 2179.29 to 4535.76 USD.
- Economically, the IMMSD required a low capital cost of 700 USD and annual costs of 996.12 USD, generating yearly savings of up to 14,015.9 USD. The payback period was under one month, proving excellent cost-efficiency.

## Data Availability

All data are provided within the article.
